# Spirit of the Foreign Periodicals, &c.

**Published:** 1842-01-01

**Authors:** 


### Spirit of the Foreign Periodicals, &c.

---

### Remarks upon French Physic and Physicians by a German.

We all know the importance of hearing the opinions of enlightened strangers on our habits, manners, and institutions; self-love and egotism are too strong among nations, as well as among individuals, to allow the parties themselves to form an impartial judgment.

Dr. *Wunderlich*, of Stuttgard, recently visited Paris, and carefully examined the numerous hospitals of that attractive capital. Being well acquainted with French literature, and having had an opportunity of coming into contact with many of its most distinguished ornaments of the present day, he has thrown his observations together in a small volume, entitled "Vienna and Paris," from which we shall make a few extracts.

"At Paris," says he, "a young physician is obliged to do a *something*, and that as early as possible, to bring himself into notice; if he does not every now and then busy himself to strike out a novel idea or discovery, on some subject or another, he will inevitably lapse into the most profound obscurity. It is of very little consequence whether the propounded discovery be soon found to be unimportant, or even utterly worthless; the momentary effect has been produced; the author is spoken of, and his fortune is made. A German, on the contrary, is amassing materials during the whole of his life; he goes on, heaping facts upon facts, and waits many a long year before publishing anything: he then may acquire the reputation of being a profound solid man, although he has never brought anything before the public."

"When a Frenchman meets with what seems to him a new idea in his path, he at once makes it known in every possible way; it is propounded and illustrated in lectures, extended in memoirs, condensed in reclamations, and finally brought again and again forward in a variety of articles by pupils, who fatigue every one that approaches them with their favourite theme. If the discovery turns out to be a fallacy, it is, as a matter of course, forgotten, and the author sets about finding out another. If, however, it is at all admitted, he at once derives honour and profit; but he does not allow himself to sleep upon it: for the public is forgetful, and he must soon seize some other plan of re-awakening its attention. In Germany a *savant* broods over an idea until it has become old, or until a more active neighbour carries it off from him. In this manner nothing, generally, is published at a mere hazard, and often nothing at all is published by the discoverer himself; hence, in spite of the immense development of German literature, works which contain new ideas are exceedingly rare. In France, the spirit of emulation (*concurrence*) gives birth to numerous abortive productions; but, at the same time, it has the effect of quickening the mental faculties, keeps them in constant play, and thus often ends in producing good results. In Germany, men of merit fall back upon themselves, repose in the conviction that they know more than other people, and are silent. There is amongst us (Germans) more than one physician whose equal for knowledge and experience could not be found in France; but unfortunately science is nothing the better for this; for the light is hid under a bushel, and the treasures are buried in the grave. How many able men shun publicity and keep all their ideas to themselves, for fear of being attacked by bold and ignorant critics!"

*Effects of Centralization.*

"In France every thing is *at* Paris and *for* Paris. Whoever has pretensions to glory or fortune goes to the metropolis; if he succeeds in dictating laws there, the whole of France receives and admits them. If they are started in any of the provinces, they inspire no confidence; for it is in Paris where are all the journals, the hospitals, the editors, and the public 'which approves or condemns without appeal.' To desire a modest success, is to desire no success at all; a man must either shine and be known, or he must be satisfied to remain a cypher. Now no wise person can fail to perceive the numerous disadvantages of this exaggerated spirit of centralization; they are admitted by many of the ablest men in France, who nevertheless strive all in their power to counterbalance and oppose it.

In Germany it is far otherwise; the multitude of medical schools diffuses a love for science among the most humble village practitioner; for near him is some university where he can refresh his knowledge and professional zeal, or communicate the fruits of his own experience.

There results, we must however confess, from this system a degree of scientific anarchy, and a fractionary division of ideas which may be injurious to the progress of general science. But this inconvenience is less prejudicial than the former. To sum up our thoughts in a sentence, we should say, that in France the despotism of the capital paralyses the insulated efforts of the original exertions of the provincial practitioners, and that in Germany they are too apt to follow a beaten path of routine."

"From the very commencement of his studies, the French student pursues a different plan from that usually followed in Germany. It is in the Amphitheatre that he begins his operations, and there he carries on his dissections with zeal and assiduity; for he well knows that knowledge of anatomy, and manual dexterity are by far the best, if not the only, means that can open a new path to him. He first becomes a candidate for the situation of *externe* in some hospital; every place and every distinction which he aims at is the prize of a first *concours*. Without doubt these public trials have their inconveniences; but they possess the advantage of obliging the student to express his ideas with clearness, and enounce them without embarrassment.

Then, in the hospitals he studies diseases at the bedside of patients, and not in books. He thus acquires a clinical tact and experience far beyond his literary acquirements, and I have known many *internes* who could quite confound the generality of German physicians in matters of diagnosis and symptomatology. In theoretical medicine, in chemistry, botany, and other accessory sciences, they are far inferior to our countrymen.

During the rest of his career, the French physician follows the same path. To acquire greater skill in diagnosis, and to excel his professional brethren in the accuracy and skill of his judgments, is the constant aim of his life. In hospital practice this point almost exclusively occupies his attention. What matters it to a Frenchman to know the best means of curing a disease? Does not pathological anatomy teach him that most diseases are incurable? All therapeutic attempts also are made with formulæ previously determined upon, without regard to the idiosyncrasy or individual constitution of the patients.

In Germany, it is unhappily quite otherwise; our hospitals are too often only places of retreat for old physicians, and in the eyes of the public it is anything but a recommendation to be attached to an infirmary. Hence the experience of the private practitioner is occupied with devising a number of trifling prescriptions, and with concocting medical theories which, from being based on insufficient foundations, are too often merely visionary and futile."

"Another peculiarity of French practice is the custom of selecting a particular branch of medicine or surgery for more especial study. Every part of the human body has its peculiar doctor. In Germany we know nothing of this sort, with the single exception of the study of mental disorders. There is abuse in the practice of both countries. The man who is generally well informed and who devotes his attention to any special branch of professional study may certainly render great services to humanity, and it is certain that it is to such practitioners that we are indebted for a number of valuable discoveries and improvements—lithotrity for example. But not a few distinguished practitioners of France, as MM. *Velpeau, Breschet, Lisfranc, Malgaigne, Rayer* and *Louis,* have shewn us that special pursuits may be advantageously combined with the most general acquirements, and that a man, who is acquainted with every branch of professional science, is far superior to the small geniuses of small specialties."

. . . . . . After some remarks on the very general ignorance among French physicians and surgeons of what is done in other countries, and the consequent conceit which they entertain of their national superiority, and after claiming for the Germans a decided superiority over their more lively neighbours in physiology, (compare the treatise of *Müller* with that of *Magendie,* and judge), Dr. *Wunderlich* offers some good observations on the attention which of late years has been paid by the French to medical statistics. While admitting that the researches of such men as MM. *Louis* and *Andral* cannot fail to be useful, he adds: "the numerical method cannot lead us to the solution of problems in pathology and in therapeutics; for, on the whole, the phenomena, which are comprehended under the same denomination, are too variable to admit of exact comparison, and if we wish to be guided by statistical data, we can never be certain that the patient under treatment does not belong to the number of exceptions rather than to the general rule. A single case attentively observed and well reported contributes more to the advancement of pathology than entire volumes full of figures. Again, in therapeutics the numerical method is of still more difficult and misleading application; and, when I hear of a French physician mercilessly (impitoyablement) dividing his typhoid patients into three classes, one set to be treated with bleeding *coup sur coup,* another with purgatives, and a third with low diet alone, I feel as if we had relapsed into those ages of barbarism when experiments were made upon condemned criminals. Such a mode of proceeding seems to me like that of an entomologist who unfeelingly lets his poor victims die with a pin stuck through their bodies. Why should we abandon the beautiful path which *Bichat* so ably traced out—a plan at once rational and philosophic. It has been by following in his footsteps that French medicine, personified in *Laennec, Bayle, Corvisart, Broussais,* and *Cayol,* has made its most valuable acquisitions. Medical statistics are on the whole a delusive means of enquiry, which should be employed with extreme reserve, for fear of its misleading by its apparent exactitude those who are not acquainted with the laws of probability in physical and moral science."

"While I willingly acknowledge the great talent for observation possessed by French practitioners, I cannot omit to reproach them with being seldom disinterested in their researches. They are always endeavouring to confirm some preconceived idea, or to sustain a theory which they have adopted. If they have gained a reputation in any special branch, or if they have acquired much dexterity in diagnosis of certain diseases, every other subject is lost sight of, and they can see nothing but their own favourite affairs: I may allude, for example, to M. *Piorry,* and his hobby-horse of auscultatory percussion, to say nothing of the pedantic nomenclature which he has attempted to introduce. Another defect in the French character is, that they rarely pronounce a prognosis until the advanced stage of a disease. M. *Bouillaud* seldom says any thing of the danger of any case, till the patient is almost moribund: he regards himself as the representative of the physiological school of medicine, and yet seems to me not to understand aright the genuine principles of it. Moreover his incessant disputes

N 2

must surely prevent him applying his subtle mind to the higher questions of our science.

MM. *Louis* and *Andral,* although given to almost over-minute observations, very fortunately for science keep themselves free from theoretical abstractions. The latter has shewn by his writings how well he has seized the spirit of physiological medicine; but on some occasions he is not quite faithful to its principles, and is apt to attach an exaggerated importance to morbid alterations. The former, although one of the founders of the numerical method of studying the phenomena, has avoided the evils which a less skilful physician would have fallen into; for, while basing his observations on pathological anatomy, he knows at the same time how to view the phenomena in a physiological point of view.

M. *Magendie* follows out the plan which he has laid down for himself, that of investigating the laws of the system by direct experiment, with great assiduity and ability. He has purged physiology of not a few speculative errors with which it was defaced; but he attaches too much importance to the results of his vivisections, and vainly endeavours to explain the mysteries of living action on mechanical principles.

M. *Chomel* is the physician of the French metropolis who pleases most the generality of foreign physicians. He has a most winning lucidity and neatness of expression, disguising often many difficulties by the graceful clearness of his language. He is an *eclectic* both in his practice and in his reasonings, and is thereby led into not a few contradictions, which are sure to be laid hold of and exposed by his indefatigable opponent, M. *Bouillaud.*

M. *Cruveilhier* classes and analyses the morbid alterations of the body with singular sagacity, which is enhanced by his remarkable talent for observation. Although duly appreciating the importance of the study of pathology for the advancement of medicine and therapeutics, he generally avoids philosophising upon any subject.

MM. *Rayer, Martin, Solon, Gibert,* and *Ricord* have shewn decided talent in investigating particular classes of diseases; but we must wait to find out whether they will exhibit as much skill in the study of more general enquiries."—*Revue Medicale.*

*Remarks.*—We have repeatedly, during the last six or seven years, expressed our opinion on the character of the modern French School of Medicine, and the readers, who have done us the honour of regularly perusing our comments on the medical literature of foreign countries, cannot have failed to notice the marked coincidence of sentiment between our observations and those now made public by Dr. *Wunderlich.* In the number of this Review for July, 1836, there is a somewhat lengthened article on the three sects of medical professors now existing in Paris—the Physiologists, the Hippocratists, and the Eclectics—with an illustration of the respective doctrines of each: we have reason to believe that our remarks were admitted by good judges to be thoroughly just and withal instructive. In the number for January 1835, we contrasted the character of the French and the German physicians in the following passage, which may not inaptly be quoted here, as an appendage to Dr. *Wunderlich's* comments.

........ "None can be more willing to bear testimony to the high merits and surpassing excellencies of the Germans in some respects than we are; for where do we meet with such unconquerable perseverance, such unwearied industry, and so ardent a love of science, for science' and not for fortune's sake, as among them?

It would seem that Nature is jealous of excessive kindness in the distribution of her intellectual favours, and that it is only on a very scanty few that she bestows the aggregate of them all. There is, as it were, a compensation-system adopted in the general award, so that, where one faculty or set of faculties is developed in an unusual degree, the superiority arising from their excellence is counteracted by the deficiency or irregularity of some other faculty or faculties. For example, compare and contrast the mind of a Frenchman with that of a German—what a striking difference! the quickness, alacrity, and lighthearted promptitude, the hop-skipping, grasshopper-like restlessness, the past-forgetting, novelty-attracting, and consistency-despising character of the one; and the patient, plodding, laborious hardihood of the other, with his fact-collecting acquisitiveness, burden-oppressed memory, and slow, self-bewildering judgment, often led astray by his active shaping ideality, just as a traveller is by the twinkling of a lanthorn-fly in a dark night.

Both of these characters have their advantages and evils; be it our part to cull the former and reject the latter......... Whether our continental brethren are led to form as unfavourable an opinion of the merits of the British medical journals, as we reluctantly are compelled to form of theirs, we cannot tell; but, with all kindliness of feeling for them, and with an entire freedom from national partiality and prejudice, we hesitate not to affirm that there is more practical information, more substantial food for the mind, more, in short, of strong, shrewd, every-day good sense in one number of any of our established periodicals, than in the annual series of many of the continental."—*Rev.*

---

### M. Andral on Alterations of the Blood, with Comments on the Old Humoral Pathology.

In the three last numbers of this Review we have given tolerably copious extracts from the lectures of this eminent pathologist on the changes, in the relative proportion of the different constituents of the blood, which are found to occur in different diseases. We have shewn that, in some, it is the fibrine that is chiefly affected; in others, that it is the red globules; and in a third set, that it is the watery portion of the blood whose quantity is either abnormally increased or diminished. In the present article we shall consider the principal alterations observed in the blood by the admixture with it of foreign matters, whether these be generated in the system itself or introduced from without. We begin with—

*The Bile.*—Although much has been written on the diseases which the introduction of this secretion into the circulating fluids has been said to induce, we must remember that its actual existence in the blood has never been demonstrated by chemical analysis. One of its constituents, its colouring principle, has been observed in the serum, communicating to it a green or a deep yellow hue: if nitric acid be added to such serum, its precipitated albumen also is usually more or less decidedly green. M. *Martin Solon,* in his work on albuminuria, has related four cases of pleuro-pneumonia, complicated with bilious symptoms, in which a green precipitate was induced in the urine, when nitric acid was added to it. After the use of brisk purgatives for a few days, the green colouring matter disappeared.

*The Urine.*—The same may nearly be said of this secretion as of the bile. It has never been detected *en nature* in the blood; but one of its elements, the urea, has been. Every one knows the interesting experiments of MM. *Dumas & Prevost,* who, after extirpating the kidneys in some animals, discovered the presence of a notable quantity of the urea in the blood. Such being the case, we might *à priori* infer that, whenever these organs perform their functions imperfectly, the urea may gain admission into the circulating fluids. Now such has been found to be the case in the *morbus Brightii,* or granular degeneration of the renal parenchyma.

*The Milk.*—Every medical man has heard of the "milk-fever," and many an old practitioner in the present day will still tell you that it is altogether owing to the introduction of this secretion into the blood. The results of chemical analyses, however, demonstrate that this notion is quite groundless. Some writers indeed have asserted that they detected the essential principle of milk, its caseum, in the blood; but the assertion is not credited by the best authorities.

What has been called a milky state of the blood has not unfrequently been noticed; but it is a curious fact that this phenomenon has never been found in the case of a puerperal woman. It is the serum of the blood that exhibits the peculiar milky appearance, that has imposed upon so many writers. MM. *Trail, Caventou,* and *Christison* have analysed such blood; but on no occasion have they ever detected any of the elements of milk in it. The peculiar appearance depends on the admixture of a certain portion of fatty matter, which forms a sort of emulsion with the serum. Such at least is the opinion of Dr. *Christison;* M. *Caventou,* on the other hand, attributes it to the presence of albumen somehow altered and modified.

We shall now notice some of those foreign matters, the products of morbid action, which have been occasionally detected in the blood. By far the most important of these is *pus.* It has been found in cases of—1, inflammation of the parietes of the heart, arteries, and veins; 2, eruptive fevers, small-pox for example; 3, large abscesses, and then the pus penetrates by absorption, molecule by molecule; and, lastly, pus has been found in the centre of sanguineous coagula, which must have been formed during life.

When purulent matter gains admission into the blood, in some cases the two fluids become so blended together that the colour of the latter is more or less changed; while in other cases it is found to be deposited in the form of minute insulated drops floating in the mass of the blood. In the former condition, the pus seems to act as a poison; for the blood is always found to have lost its normal consistence, its solid portion being unusually soft, friable, and broken down into small clots, and its serum being a veritable ichor. When pus and fresh-drawn blood are mixed together, the latter is found not to coagulate; as we have on a former occasion shewn to be the case. The same condition of the blood, its uncoagulability, is induced by the introduction of various poisonous substances, whether of a vegetable, animal, or aerial nature, into the system. How these agents act is not at all understood; but the fact of their doing so in the manner alluded to has been known from the earliest days of medical science. M. *Magendie* has for some years past been prosecuting a series of experiments to illustrate the effects of the introduction of foreign matters into the blood in the production of disease; and he tells us that he can, at will, induce all the symptoms of putrid fever in dogs, &c., by injecting into their veins a putrescent animal fluid. We doubt however whether this be the right mode of arriving at any satisfactory conclusions.

"I have now finished," says M. *Monneret,* the reporter, "the accurate description of those important researches undertaken by MM. *Andral* and *Gavarret.* The question as to the nature of fevers, a question that has been so long agitated, has now received, not indeed a complete solution, but a bright light, which cannot fail to render many points connected with their history much more clear than they have been hitherto. It can no longer be admitted that any one should now assimilate typhoid fever with a genuine inflammation or phlegmasiæ; the marked differences as to the state of the blood in these diseases establish a well-defined demarcation between them. The partisans of the Broussaian school have not been satisfied with M. *Andral's* analyses of the blood, because they are not in harmony with their doctrines; but this mode of objection cannot be received, and the only way to overthrow his statements is to go over the ground which he has so diligently traversed." . . . . . . . "The alteration of the blood takes place very early in eruptive and typhoid fevers, and becomes more and more decided as the disease advances. We may confidently affirm that the change in the quality of the circulating fluid precedes, by a considerable period of time, any lesion of the intestinal mucous glands—the primary *point de depart* of all the phenomena of fevers, according to *Broussais* and his followers.

Let it be remembered that we do not mean to say that the change in the composition of the blood, although it is unquestionably anterior to the lesion of the Peyerian glands, is the *cause* of fevers; it is rather one of the earliest effects. We have not yet succeeded in detecting the primary link in the chain, and perhaps we never shall." (The doctrine of the old physicians, that the miasmatic poison of genuine fevers acts primarily on the nervous system, and subsequently on the blood, is probably the correct one.)

. . . . . . . . "However important the study of the blood is, we must be careful not to separate it from the examination of the solids. *Bichat*'s memorable saying, 'that every exclusive theory, whether of humorism or of solidism, is a pathological absurdity,' should never be forgotten. The great aim of the wise physician will be to strive to ascertain what share each of the two great elements of all organization takes in the production of diseases. Morbid anatomy has revealed a numerous catalogue of lesions of the solids; but, as it has omitted to examine the changes of the fluids, the aid of analysis should now be called in to lead us to the truth." . . . . .

*The Importance of the Microscope.*—"The microscopic examination of the fluids and solids is a most valuable part of morbid anatomy, as well as of morbid chemistry. Already it has conferred great benefits on medical science, and still more important discoveries no doubt are yet to be made. The scalpel, the test-tube, and the microscope are the three equally necessary implements of pathological study,—and the right mode of using each of them should be well understood by every medical student. Hitherto the former has occupied an almost exclusive attention; but now a sounder method of examination has been introduced, and already has produced some valuable results."

*The Old Humoral Pathology.*—Before dismissing the subject of alterations of the blood, M. *Monneret* has very appropriately re-called to his reader's attention the opinions of one of the physicians of the last century, Dr. *Huxham,* on the influence which such alterations have in the production of fevers and other diseases, with the view of comparing them with the doctrines of the modern humoral pathology.

*Huxham* commences his account of fevers with a description of simple fever or synocha, which, he says, is produced by an increase in the action of the solids on the fluids of the body, and by the subsequent re-action of these upon the former. He mentions three cases in which this morbid state may be induced. The *first* is that of a person who has taken any violent exercise; here the mere excess of the action and re-action of the solids and fluids on each other is sufficient to bring on the state of simple fever. In the *second* case, the quantity of the fluids is increased; as for example, by the sudden check of the perspiration, when this is abundant: fever is the consequence, and nature makes an effort to get rid of the excess of the humors. The *third,* is that of a person who has drank a large quantity of wine or any other stimulating fluid; the excitement brings on a simple fever, which, however, will quickly yield to rest and abstinence.

Sometimes the blood is driven along with so much force that its globules penetrate into those minute vessels which do not usually admit them; hence the obstructions and congestions which so often occur in the course of some fevers. The chief remedial agent recommended by *Huxham* is bloodletting,

"which by diminishing the quantity of the red globules renders the moving force more feeble."

Now this idea, that the detraction of blood has the effect of diminishing the number of the red globules, has been found by the researches of MM. *Andral* and *Gavarret* to be quite correct; and it is curious to observe that the results of recent analyses are in perfect harmony with the assertions of the old pathologists,—assertions, too, based upon mere speculative theories. We have already stated that in plethora there is always an increase in the quantity of the red globules, and that bleeding is the appropriate remedy for this condition; seeing that it very quickly diminishes the quantity of these, and does not very sensibly affect the proportion of the fibrine for some time. At the period at which *Huxham* wrote, considerable attention was paid to the microscopic examination of the fluids, in consequence of the great interest that had been excited by the researches and discoveries of Leuwhoeck.

He admits three leading kinds of alteration of the blood; the two first are what he calls the constitutional states of the blood, and the third is that of its dissolution and putrefation.

The first abnormal modification is that in which the red globules are increased in quantity, squeezed one against the other, and pressed too compact and dense; hence, he says, arises an over viscid state of the fluid, and an unusual tendency to coagulate and solidify, when drawn from the body; at the same time the increased rapidity of the circulation causes an unusual degree of friction of the blood against the sides of the vessels, causing a greater development of heat. When such is the case, there is always a disposition to synochal fever, and to obstructions in the minute vessels. He attributes the buffy coat to the heat of the fever, which tends to coagulate the serous portion of the blood, and to convert it into a jelly.

We need not say that the theoretical edifice constructed by the English physician with so much ingenuity crumbles down before the recent analyses of the blood: as to the notion of the greater friction of the globules against the walls causing a greater evolution of heat, we all know that it is quite conjectural, and far too mechanical to be true.

2. The second morbid constitution of the blood is characterised by the diminution in the quantity and in the density of the globules, and by an increase in that of the serum or watery portion. This condition is accompanied with pallor of the surface, great weakness, imperfection of the secretions, and a tendency to dropsical effusions. There is also a disposition to obstructions, not indeed of an inflammatory kind, as in the former case, but of a cold or torpid character from the diminished excitability of the vessels, in which the blood is apt to stagnate. Passive congestions too are liable to take place in this manner. The diminution in the proportion of the red globules, which *Huxham* had adopted on hypothetical grounds, has been demonstrated in the present day. This condition is observed in cases of anæmia, chlorosis, and of cachexia induced by long continued chronic diseases.

3. The dissolved state of the blood is observed in scurvy, which may be considered as the type of this alteration: hæmorrhage and adynamia are the chief phenomena indicative of its existence. The coagula of the blood are soft, loose, diffluent, and without any buffy coat. The serum separates only imperfectly from the coagulum. The blood drawn from persons affected with petechiæ and ecchymoses often forms a uniform semi-coagulated mass of a darker colour than usual, and passes readily to putrefaction. *Huxham* believed that the hæmorrhages are generally owing to the acrimony of the fluids destroying the contexture of the blood itself and corroding the minute capillary vessels. He says that, if we examine the blood while within the vessels, we may observe the globules elongated to enable them to traverse the small tubes. He thence infers that they become broken up in their passage and that their debris enters readily the excretory vessels and transudes from their extremities. He thus accounts for the hæmorrhage which not unfrequently occurs from the bowels and urinary passages, as well as for the petechiæ and ecchymosed spots on the skin and some of the mucous surfaces. A dissolved state of the blood is not incompatible with the development of inflammation. *Huxham* describes particularly an epidemic pneumonia which was exceedingly fatal, especially among the inmates of prisons, ships, &c. and in which, although all the symptoms of the disease of the lungs were present, the blood drawn was soft and devoid of its normal consistence. The causes of this condition of the blood are, according to him, two fold. In some cases it is the result of spontaneous changes within the vessels, and in others it is induced by the introduction of poisonous matters into the system. Among these—the poisonous matters—he enumerates alkaline salts, muriate of ammonia (which has the effect of destroying or dissolving the blood-globules very rapidly), cherry-laurel water, mercury, salt and decayed provisions, the virus of serpents, &c. An elevated temperature has been considered by some to be sufficient to induce a dissolved state of the blood. *Boerhaave* tells us that he confined a dog in a heated stove, and that, in proportion as the animal sweated profusely, he observed a tendency to hæmorrhage from various parts of the body. However we may view this experiment, it will be admitted by all that fevers, in which the blood is in a thin uncoagulable state, are more common in hot than in cold and temperate climates.

As to the *intrinsic* causes, or those which are generated within the system itself, we may allude first to the admixture of purulent and other septic matters with the blood. The recent experiments of *Gaspard*, confirmed as they have been by subsequent researches, have most satisfactorily demonstrated the striking changes which the blood undergoes by the contamination of such matters. They seem to act more especially by modifying the relative proportions of the fibrine and the red globules, either by diminishing the quantity of the former or by increasing the quantity of the latter.

In taking leave of *Huxham*, we cannot avoid expressing our admiration at finding in his writings such a multitude of curious facts which, far from having been overthrown by the discoveries of modern analysis, have been confirmed in a very striking manner by them; and our admiration is the greater when we remember that it was only by reasoning and hypothesis that he was led to the enunciation of such important truths.—*Gazette Medicale.*

---

### On Gangrenous Affections in the Puerperal State.

The mode in which gangrene takes place, and the causes which give rise to it, are often exceedingly obscure, except indeed in those cases in which it can be attributed to a direct obstruction of the circulation, as from closure or disease of the bloodvessels, or from mechanical constriction of any of the tissues of the body. Formerly the difficulty was imagined to be at once explained by summarily saying that gangrene is one of the consequences of inflammation. In the present day, on the contrary, some authors have gone so far to the opposite extreme as to think that, even in those cases where inflammation precedes the occurrence of the death of a part, this latter lesion is not the necessary effect of the former; but that it is connected with it accidentally, either from some obstruction to the course of the blood produced indeed by the inflammatory action, or from the introduction into the system of some foreign substance, such as air or some poisonous matter, or lastly, from a peculiar morbid state of the constitution itself.

Struck with the frequency with which gangrene takes place in the course of puerperal diseases, M. *Reynaud*, in his recently published memoir on this subject, has brought together many cases in illustration, and after pointing out the fluctuating and ill-supported opinions of most writers—opinions which are almost always based on the doctrine of gangrene being the result of inflammation or of putridity—he groupes them in two classes:—1, gangrenous affections attributable to inflammation, and 2, those attributable to infection.

As to the cases reported in the first class, we might have expected to find that in them there had been present all, or at least the most prominent, signs of inflammatory action, developing itself gradually with greater or less rapidity, and ultimately terminating in gangrene; and yet this is not the case, if we may judge from the details narrated in the reports. Thus, in the first case, which is headed *puerperal peritonitis with gangrene of the abdominal parietes, peritoneum, and intestine*, the gangrene appeared, on the eleventh day after accouchement, on the surface of the abdomen, without having been preceded by any redness or swelling of the part; and it is not stated in the dissection of the patient, who died at the end of the third week, that the gangrenous portion of the abdominal parietes exhibited any traces of pre-existing inflammation.

The author narrates among the same groupe several cases where death occurred a few days after laborious delivery, and in which the internal surface of the uterus exhibited a gangrenous eschar of some lines in thickness. In these cases he attributes the lesion to the violent manœuvres which the body of the womb had sustained. But, when the uterus has been in part disorganised by the efforts which a difficult labour has rendered necessary, should we attribute the gangrene in such cases to inflammatory action? Is it not rather a result altogether mechanical, such as we observe to take place after severe contusions, in which inflammation has little or no part at all?

The author seems to have been himself aware how little the phenomena of inflammation can be supposed in such cases to have any thing to do with the development of gangrene; as he tells us that, in these circumstances, we must not forget to take into account the condition of the female system after labour, or, in other words, the puerperal state. On the whole, we are inclined to give it as our opinion, judging from the very cases which M. *Reynaud* has recorded in his first class, that inflammation has only a very doubtful part in the production of gangrene in puerperal women; at the same time we acknowledge that it often accompanies it, as if the two states, gangrene and inflammation, were the result of the same cause. This leads us to the consideration of the *second* set of cases, those of gangrenous affections induced by infection.

Puerperal diseases arising from infection differ from other diseases attributable to the same cause in this particular, that in the latter the infection usually proceeds from without, while in the former it is engendered in the bodies of the patients themselves: such is at least the opinion of the physicians of the French school, who entirely reject the doctrine of the transmission of puerperal affections by direct contact.

Whatever may be the origin of the infection, we must not the less keep in remembrance that, occasionally under the influence of certain changes in the fluids of the body, we observe gangrene taking place. It is true that if we try to push our enquiries further, and to discover what it is that constitutes infection, or how it acts in producing gangrene, we find ourselves quite at a loss; but it is one step, and that an important one, gained, to have ascertained that such cases are of too frequent occurrence to be regarded as merely the results of a simple coincidence.

In this point of view, the work of M. *Reynaud* is very interesting, although many of his observations are far from being satisfactory. What connexion, for example, can be found between the external veins filled with pus, and pulmonary gangrene, or between even inflammation of the lymphatic glands and gangrene of the uterus? None, certainly, as far as we can trace; and yet we do not deny positively that these different morbid states may not be connected with each other. We are disposed to attach more importance to another source in explaining some of the facts adduced by M. *Reynaud*, in illustration of the tendency in the female system after delivery to gangrenous affections—we mean, the absorption of unhealthy secretions from the internal surface of the womb, and their introduction into the circulation, first of the uterus, and ultimately of the entire system.

The ingenious explanation given by M. *Genest* of the development of pulmonary gangrene in cases of apoplexy of the lungs and of metastatic abscesses in their parenchymatous tissue, by the admixture of the external air with the blood or purulent matter, should certainly not be lost sight of in investigating the phenomena of certain puerperal diseases.

We are glad to observe, from a recent account of the different forms of puerperal fever observed at the Hôtel Dieu in Paris, during the year 1840, by M. *Bourdon*, that this intelligent physician has shaken off the trammels of the Broussaian school as to the proximate cause of the disease:

He says, "The fever exhibited always the same characteristic symptoms, with a peculiar aspect and march truly remarkable; while the lesions found on dissection were very different in different cases. Several of the lesions might be attributed to the inflammation, which, in this disease, terminates with extraordinary rapidity and ease in the formation of purulent matter in different parts of the body, such as the posterior parts of the limbs, the sub-peritoneal cellular tissue, &c.; but we could not regard certain other serious lesions, as the consequence of this peculiar kind of inflammation."

If, at the same time, we call to mind the fluid state of the blood in the heart and large bloodvessels, the softening of almost all the viscera, &c. are we not authorised to regard puerperal fever as a disease which is intimately connected with some change or poisoning of the blood?—*Gazette Medicale.*

*Remarks.*—With many of the preceding observations we heartily concur; and rejoice to find that, in France, as well as in our own country, some of the most practical writers of the day are beginning to pay attention to the state of the fluids in disease, more especially in malignant fevers. The doctrine that puerperal fever is in all, or even in most, cases an essentially inflammatory disease, and, therefore, that it requires an active antiphlogistic treatment, is fraught with the most pernicious consequences. The capital error that has been committed by most writers arises from the opinion, that this fever is at all times and in all seasons of the same *type;* whereas, in truth, perhaps no two epidemics of the disease are alike; just in the same manner as the epidemics of typhous and of the exanthematous fevers are observed to vary exceedingly in different years. We have too long forgotten to take into our consideration of such diseases the influence of which *Sydenham* and many of the older writers have denominated the *medical constitution of the season;* and yet what practical physician can have failed to observe the striking difference in the general character, as well as in the mortality, of febrile disorders in different seasons? Take, for example, scarlatina; is it not sometimes so mild as scarcely to require any medical treatment at all? whereas during another year it is attended with high phlogistic symptoms, and in a third it exhibits the type of malignancy and putridity.

The same holds good of puerperal fever. It is quite true that in some epidemics, and, we may add, in some cases during all epidemics, the disease is essentially inflammatory; but it is equally true that in other cases the inflammatory symptoms, if they occur at all, are only added to, and, as it were, grafted upon a morbid state of the system, which is dependent upon a vitiated state of the fluids, and a consequent serious lesion of the entire nervous system. Now this is very nearly the doctrine of the older school; and, however humiliating to modern pride it may be to find that with all our improved methods of research we are in a great measure coming back to the long obsolete opinions and practice of last century, it is only wise and right that we should do so when convinced of our errors.

We cannot close these few remarks without recommending to the especial notice of our readers the admirable Treatise on Puerperal Fever by Dr. *Ferguson*, published two years ago, and of which a copious review was given in our number for April 1839. One very short extract will enable them to judge of the spirit of its contents.

"The three following propositions embody my views of the source and nature of puerperal fever.

1. The phenomena of puerperal fever originate in a vitiation of the fluids.
2. The causes, which are capable of vitiating the fluids, are particularly rife after child-birth.
3. The various forms of puerperal fever depend on this one cause, and may readily be deduced from it."—*(Rev.)*

---

### CASES OF GLANDERS, WITH REMARKS.

Dr. *Delaharpe*, physician of the hospital at Lausanne, has published in the Revue Medicale of Paris a memoir on this curious pathological subject. We shall first briefly report two cases which he adduces, and then extract some of his most interesting remarks.

An old pedlar was admitted, on the 17th of August, 1837, in the following state. There was extreme prostration of all the vital powers, and a tendency to delirium, although the patient, when spoken to, answered questions with tolerable accuracy. His face was puffy and of a purplish red colour; the left side of the nose, and the adjoining part of the face, were covered with numerous pustules of different sizes, each one being surrounded with a livid areola, and filled with pus. The largest of these pustules had an indurated base, and resembled a good deal the pustules of variola in suppuration. The left eye was completely closed by the swelling of the eyelids; but, on opening these, the conjunctiva was found to be in a state of *chemosis*. There was a discharge of puriform mucus from the nostrils. On different parts of the surface, but chiefly on the extremities, pustules, similar to those now described, were scattered up and down; and at the side of these pustules were observed patches of a livid hue, and also rounded soft subcutaneous tumors, some of which were of the colour of the surrounding skin, and others were slightly inflamed. The largest of these tumors gave a distinct sense of fluctuation under the finger; several of them looked like large warts, or rather like some of the forms of *nævi*. On the left buttock was a large gangrenous ulcer, which discharged a fetid ichor; the scrotum was œdematous, of a blueish colour, and exhibited several ulcerated pustules on its surface; its epidermis had become detached in some points, apparently by the friction of the parts.

The breathing was hurried, and incommoded a good deal by the obstructed state of the nostrils: the pulse was rather quick but full; the lips and teeth were covered with a dark coating; the tongue was red at its tip, but furred behind. This patient died on the following day in a comatose state.*

*Dissection.*—On removing the integuments of the face, a multitude of small abscesses were observed, varying in dimensions from the size of a small nut to

---

* No satisfactory information could be obtained whether this man had ever any thing to do with glandered horses. It was found, however, that he had often been in the habit of sleeping in stables.

that of a millet-seed; all of them contained a yellow thick pus. The left malar bone was denuded at one point, where a pustule was situated. The nasal fossa on this side exhibited large reddish fungosities, which were traced upwards to the ethmoidal cells, and backwards to the pharynx; the nasal passage was completely obstructed, and the natural tissues were with difficulty recognisable amidst the fungous mass. On detaching the tumefied mucous membrane, a multitude of minute pustules were brought into view. The posterior edge of the velum palati, at the junction of the two pillars, was found to contain a small abscess full of matter. In the right nasal fossa, the inferior spongy bone exhibited a fungous patch similar to what, as we have described, was observed in the left one, but only much smaller.

There was considerable subarachnoid effusion on the convex surface of the *brain;* but the encephalon did not present any other decidedly morbid appearance. The lower half of the *trachea* was inflamed, swollen, and its mucous membrane a good deal softened. Here and there were observed minute abscesses, similar to those found in the nasal fossæ. The *lungs* were healthy throughout, with the exception of a minute abscess in the upper part of the right lobe. The heart was normal; so also was the liver; the spleen was friable and softened.

*Case* 2.—A postillion, 33 years of age, was admitted on the 10th of November 1835, into the hospital at Lausanne, in consequence of severe pains in the lower limbs, and more especially in the right knee; there were also several painful rounded tumors on different parts of the limbs. One of these, situated beneath the right knee, was of the size of a large egg, and communicated to the finger a distinct sense of fluctuation. A similar tumor, but of smaller dimensions, existed on each arm. There was slight hydarthrosis of the left knee-joint. The patient was put on a nutritious diet, and he was ordered to drink a decoction of mezereum and other woods. One of the tumors on the left leg was punctured with the lancet; but nothing save a little dark blood was discharged from the wound. The other tumors were kept wetted with a decoction of oak-bark, to which some spirit of wine had been added. For three months this patient continued in a weak and ailing state; but, by persevering in the use of tonics, wine, and a generous diet, his health and strength began to be so much improved that he was able to leave the hospital in the following March; he died however during the course of the Summer of marasmus.*

The disease of glanders is communicable to the human subject by atmospheric infection as well as by the direct inoculation of the diseased matter by a wound. The observations of Dr. *Farozzi* of Milan in *Omodei's* journal for August 1822, prove this. *Hufeland* seems to have been the first to pronounce that the disease in the horse is communicable to man: in a note to a case of doubtful character reported in his journal for March 1822, he exclaims, *der rotz der pferde steckt menschen an,* (the glanders of the horse infects men)! Veterinary surgeons had long known that wounds of the fingers, if infected with the nasal discharge of sickly horses, are apt to assume a most unhealthy character; but they do not

* It is more than doubtful whether we can regard this case as one of glanders in the human subject, or *equinia,* as it has been denominated by some writers. That there was that peculiar cachectic state of the system, in which there is a tendency to the formation of subcutaneous abscesses, is manifest; but it does not appear that the disease was induced by any infection from the horse. It seems to us to be taking too limited a view of this and similar cases to refer them all to such a morbific cause. There is evidently a striking analogy between cases of this sort and the ecthyma cachecticum described by *Willan* and *Bateman.* —*Rev.*

seem to have been aware that the system under such circumstances is liable to be poisoned by the introduction of a virulent matter into the circulation. This fact, which has been clearly established of late years, cannot now be disputed by any one; although it is probable that some cases, which have been recorded as examples of glanders in the human subject, are of questionable authority. The following one well deserves to be generally known, and we shall therefore give its particulars at some length.

*Case* 3.—A man, 42 years of age, who had had charge of a glandered horse for a length of time, happened, one day before dressing the animal, to prick his finger; a few days subsequently the puncture became inflamed and painful, and the whole hand was swollen. M. *Thierry* was consulted; he ordered poultices to the finger and cooling medicines to be taken inwardly. In the course of a week, as a distinct feeling of fluctuation could be perceived in the site of the wound, a deep incision was made there, and gave issue to a quantity of purulent matter mixed with a bloody serosity. This was on the 14th of September; on the 22nd a painful swelling formed on the middle of the fore-arm; this gradually proceeded to suppuration, and, when opened with the lancet, gave vent to a large quantity of unhealthy pus. In the course of another week a second abscess formed nearer the wrist, and was treated in a similar manner. By the use of a generous diet, the patient seemed to be recovering his health and strength; but the amendment was only temporary. In the beginning of November a new abscess made its appearance on the upper part of the same fore-arm, and another about the middle of the arm of the same side: both were exceedingly painful, and the almost constant suffering of the patient brought on a feverish irritation of the whole system.

Fatigued with almost continued suffering for three months, and weakened and emaciated by the profuse suppuration from the abscesses, the patient now applied to M. *Amussat*, but without explaining to him the probable cause of his cachectic condition. This eminent surgeon suspected a scrofulous taint of the system, and prescribed accordingly. Benefit was derived from the treatment recommended, and by the 20th of December several of the abscesses had cicatrised. On the 4th of January, the two tumors on the arm and fore-arm suddenly vanished, but in the course of the same day a painful swelling made its appearance on the outer part of the right thigh; the system at the same time was affected with feverish chills and heats. This swelling gradually increased in size, and became more prominent and shining on the surface: after the ineffectual use of discutient remedies a blister was applied in the hope of dispersing it, but without any good effects. Although it had now existed for upwards of six weeks, no fluctuation could be detected, nor was this distinct until the first week of April. An incision being made into the most prominent part, a large quantity of thick and dark-coloured blood flowed out, but without the admixture of a single drop of purulent matter. This was a most unpleasant circumstance, and naturally suggested the possibility of the presence of an aneurismal sac; but, as there had not been any pulsation in the tumor, and as the discharge gradually ceased after some time, the fears of the medical attendant subsided.

A probe introduced into the wound could be made to pass for nearly eight inches in a direction right inwards and downwards. M. *Breschet* was now consulted; after listening to the details of the case, he at once pronounced that it was one of constitutional infection induced by the poisoning of the wound on the finger from the glandered horse. The venous or lymphatic system, participating in the general infection, might probably be the channel of the morbid virus being deposited in different parts, and thus exciting erratic suppurative action. The patient was taken into the Hôtel Dieu on the 4th of May, and there M. *Breschet* made an incision through the integuments along the entire length of the swelling, to the extent of nearly eight inches. On separating the edges of the wound, it was now found that the sanguineous collection, that had been discharged five weeks before, was situated in part on the outside and in part beneath the aponeurosis of the thigh. M. *Breschet* directed that the internal surface of the tumor should be wetted daily with a solution of the ioduret of potassium. A day or two after the patient's admission into the hospital, he experienced a severe pain about the middle of the front of the other thigh, where an inflammatory swelling, similar to the one already described, gradually was developed. It was laid open on the 30th of June; but this was scarcely done, before another painful tumor made its appearance below the knee of the same side, and it was necessary to make an incision into it on the 7th of July. It was gratifying however to observe that the immense sac on the left thigh had been gradually cicatrising, and every thing on the whole was going on pretty well until the 15th of this month (July), when the left ankle began to be swollen, red, and painful, and the general strength of the system became greatly debilitated. At the same time a cough, night sweats, &c. made their appearance; and diarrhœa soon followed. Towards the end of August, the right eye-lid first, and subsequently the forehead and the entire scalp, became affected with an œdematous swelling, and, a few days afterwards, a dark gangrenous spot was observed about the middle of this puffiness: this spot gradually increased, extending over the whole of the upper part of the face and entirely covering the nose; the epidermis was detached in several places, and a gelatinous bloody fluid exsuded from under it. There was also at the same time a copious discharge of viscid fœtid matter from the nostrils; delirium came on during the night; and a number of livid pustules made their appearance upon the surface of the chest and extremities. On the 6th of September the patient died.

The *dissection* was performed with the greatest care in the presence of MM. *Breschet*, *Amussat*, and many other medical men.

Without mentioning the phenomena observed in the various external abscesses, which are very minutely reported by the narrator, we proceed at once to describe the state of the nasal passages. The whole of their mucous surface was coated with a gelatiniform sanies; when this was removed, the surface appeared irregularly furrowed by ecchymoses which occupied more than half its extent, and in which could be observed several minute pustules, analogous, except in size, with those on the surface of the body: with the aid of the microscope it was found that some of them exhibited small circular points of ulceration.

The *lungs* exhibited numerous circumscribed points of lobular inflammation; the state of the heart is not mentioned.

It deserves to be mentioned that the horse, from which the patient, it is believed, caught the complaint, was examined by a distinguished veterinary surgeon, M. *Bouley*, and pronounced by him to be affected with " morve chronique dite tuberculeuse."

*Remarks.*—The most remarkable phenomenon in the preceding case was certainly the successive transference of the matter which engendered the disease upon a point sometimes remote from, and at other times more near to, the place where it had at first fixed its seat: this sort of metastasis occurred no fewer than ten times in the course of one year. There appeared a swelling, which was painful for the first few days, but without exhibiting a decided inflammatory aspect; its progress was slow; and, when it was opened with the knife, a quantity of matter, sometimes of a healthy character and sometimes more or less blended with blood, was discharged, and this discharge continued for some time. Then all on a sudden it ceased entirely, and almost invariably a new swelling made its appearance in some other part.

Another peculiarity of this case is that there were none of the symptoms of acute glanders until near its close; then the eruption of pustules on the body, the discharge of fœtid mucus from the nostrils, and the development of acute irritative fever, were not to be mistaken. But it is to be remembered that the disease is not unfrequently observed to follow nearly a similar course in the horse; the health of the animal may be for a long time but little, if at all, affected; and, were it not for the oozing of the viscid mucus from the nostrils, it might be pronounced to be perfectly sound. Suddenly, however, after this local complaint has continued for some time, the symptoms of the acute form of the disease make their appearance and the animal is quickly carried off.—*Revue Medicale.*

---

### MOST OBSTINATE HICCUP CURED WITH QUININE.

A countryman was seized, while recovering from an attack of ague, with a convulsive hiccup which recurred every few moments. A variety of antispasmodic remedies, such as ether, valerian, musk, assafœtida, opium, &c. were tried, but without relieving the troublesome symptom: blisters, also, as well as other cutaneous irritants, were applied with equal unsuccess. For nine days the hiccup continued with but little intermission, and this was only at irregular intervals for about a quarter of an hour at most.

Suspecting that, although all the other symptoms of the ague had ceased for some time, this convulsive affection of the stomach might somehow be connected with it, the medical attendant administered a large dose of quinine in an enema. A few hours after it was given, the hiccup ceased almost entirely; but it again returned, as violent as before, next day. The quinine was again ordered, and with equally happy effects; and by persevering in its use for a few days, the symptoms did not return.—*Journal des Connois. Med. Chir.*

---

### WALNUT-LEAF TEA, A GOOD REMEDY IN SCROFULOUS COMPLAINTS.

Professor *Negrier* of Angers, a respectable authority, has for some years past been trying the effects of an infusion of the leaves of the walnut-tree in a variety of scrofulous maladies, and the results of his experience have led him to form a most favourable opinion of it as an antiscrofulous remedy.

He reports a great number of cases of disease of the lymphatics, with or without ulceration of the integuments, of scrofulous ophthalmia, of affections of the bones and periosteum, &c. in which decided and very marked benefit was obtained from a course of this simply prepared tea. A handful of the fresh or slightly dried leaves may be added to a pint of boiling water, and of this infusion a small cupful may be taken twice a day. An extract may also be prepared by evaporation, and this Dr. *Negrier* recommends to be given at the same time, either in the form of pills or of a thick syrup. A strong decoction of the leaves he has used with excellent effects as an application to scrofulous ulcers.—*Archives Generales.*

*Remark.*—We know nothing practically of this remedy, but somehow or other we feel inclined to predict that it is one deserving notice, and that it will be found useful in some cases of lymphatic and cutaneous disease. Whenever we pass a walnut-tree, and smell the peculiar odour which it gives out, the idea always occurs to us that it is not destitute of tolerably active medicinal properties. Moreover, as a general remark, scrofulous diseases are, on the whole, much more benefited by vegetable remedies than by those of a metallic nature, except, perhaps, steel; and this we know is more *kin,* so to speak, to the body than any other of its class.—*(Rev.)*

### M. Cruveilhier on the Sounds of the Heart: extraordinary Case of Congenital Malformation of this Organ.

A case, unique, we believe, in the annals of medicine, recently occurred in Paris; and, as it afforded a most favorable opportunity of examining the sounds and movements of the heart, it is most fortunate that so accurate an observer as M. *Cruveilhier* has detailed the particulars at great length. He was called to see the infant, a few hours after birth, in whom the following extraordinary malformation of the chest existed. The heart, which was vigorous and acted strongly, was situated fairly out of the chest, from which it had passed through a circular opening in the upper part of the sternum; this opening had the appearance of being moulded upon its pedicle, so to speak, formed by the large vessels which proceeded from and towards it. The heart was thus as completely exposed, as if the sternum had been removed and the pericardium opened. It was of a pale colour, as if it was still covered with its fibrous envelope, and its surface was dry. It moved about by any change in the position of the body; thus, when the infant was held up, the heart, obeying it natural gravity, fell down in front of the sternum; and then the vessels, serving as the pedicle of the swelling, were dragged somewhat lower than they had been before, so as to hang in front of the perforation. Whenever this was done, the movements of the heart were hurried, and the child began to scream, evidently from the uneasiness experienced; for no sooner was it again placed on its back, than it seemed to be easier and ceased crying. The axis of the heart was nearly vertical, and not oblique as in the healthy state. Merely touching it, or even gently pressing it with the finger, did not disturb its action, nor appear to distress the child. The ventricular portion of the organ was essentially formed by the left ventricle; the right one looked as a mere appendix, and did not contribute to the formation of the apex. The auricles were very imperfectly developed; they seemed like small wings at the basis of the heart, jutting out a little on each side. To see them distinctly, it was necessary to turn the heart over; but doing this always caused the infant to scream out.

*Movements of the Heart.*—1. The contraction of the two ventricles was simultaneous and isochronous: so likewise was that of the two auricles.

2. The contraction of the ventricles coincided with the dilatation of the auricles, and with the propulsion of the blood into the arteries. The dilatation of the ventricles coincided with the contraction of the auricles, and with the *resserrement* of the arteries.

3. There were only two periods in the movements of the heart; no period of repose, as described by most writers, could be perceived. The period of contraction immediately followed that of dilatation, and vice versâ.

4. As to the question which has been raised respecting the order in the succession of the heart's movements—viz. whether the contraction of the auricles precedes that of the ventricles, as most authors believe, or the reverse—it seems to me to be nonsense; the alternate contraction and dilatation of the auricles result from two opposed forces always in action, which succeed each other in an invariable succession, after the manner of the two alternate movements of an adjusted pendulum.

5. The duration of the contraction of the ventricles is double that of their dilatation. If we divide into three equal periods the entire duration of the ventricular systole and diastole, we find that two belong to the former and only one to the latter act. The period of repose, spoken of by most writers, has been taken for the first period of the systole. The same remark applies to the movements of the auricles; their contraction occupies twice the time of their dilatation.

6. During their contraction, the ventricles become pale, their surface furrowed, and as it were shrunk or shrivelled: the superficial veins swell; the *columnæ carneæ* of the right ventricle are to be perceived; and the circular fibres (les fibres tournoyantes) of the summit of the left one, which alone forms the apex of the heart, become more distinct.

7. During their systole, the ventricles are contracted in every direction; and, if their shortening is the most obvious phenomenon, this is attributable only to the circumstance of the vertical diameter being the greatest: at the same time the apex of the heart describes a spiral or corkscrew-like movement from right to left and from behind forwards.

8. It is to this spiral contraction, which is slow, gradual, and as it were successive, that the forward movement of the apex of the heart and consequently its percussion against the thoracic parietes are owing; the systole of the ventricle is not accompanied with any distinct propulsion of its apex forwards, as is usually supposed.

9. The diastole of the heart takes place in a quick, and, as it were, instantaneous manner; it is so rapid and energetic, that it seems at first sight to constitute the organ's active movement; and it is so strong that the hand pressed firmly round it is forced open with violence.

10. The dilatation of the ventricles is accompanied with a propulsive movement of the heart downwards; it was most strongly marked when the infant was placed in a vertical position. This propulsive movement was so striking that at first I imagined that it was during the dilatation of the ventricles that the percussion of the apex against the walls of the chest took place. This idea had formerly existed in my mind from some observations made on frogs;* but, on a more attentive examination, I convinced myself that I was mistaken, and that it was really towards the close of the ventricular systole that the percussion actually occurred.

11. The diastole of the auricles is quick and *brusque* like that of the ventricles; but its duration is marked by the duration of the ventricular systole; their contraction is on the contrary as brief as the dilatation of the ventricles.

12. During its dilatation, the right auricle seems almost ready to burst, from being so much distended and its walls being so thin. The left one, being thicker, narrower, and more elongated, does not exhibit the phenomenon in the same degree.

*Sounds of the Heart.*—When the ear was applied against the heart, either directly or with a piece of fine linen interposed, the double or tic-tac sound was at once perceived. The first sound was much more feeble than it is usually when heard through the thoracic parietes. It was therefore evident that on the one hand the cause or seat of the double sound was resident in the heart itself, and on the other hand, that the first sound was aggravated or rendered louder by the walls of the chest. Let it not be imagined that the feebleness of the first

---

* We may mention here that the same idea suggested itself eight or nine years ago to ourselves, while examining the actions of the frog's heart, when laid bare, with the late Dr. *Hope*. It certainly seemed to us that it was during the act of dilatation of the ventricles that the apex of the heart was thrown forwards; and we remember pointing out the circumstance to Dr. *Hope* and some other gentlemen present: they however were unwilling to admit it, in consequence, as we thought at the time, of their preconceived opinion to the contrary. It is possible that we may have alluded to this idea—of the pulsation of the heart being simultaneous with its dilatation—in some of the former numbers of the *Medico-Chirurgical Review;* but we cannot call to mind the exact number.—*Rev.*

sound was, in the extraordinary case now under consideration, at all owing to any feebleness in the heart's movements; on the contrary, these were very vigorous, and the child appeared altogether extremely lively.

2. The loudness of the tic-tac gradually increased from the apex towards the base of the heart, and vice versâ; from this it follows that it was at the base of the heart that we had to seek to discover its cause or actual seat.

3. If the finger was applied upon the origin of the pulmonary artery—which is, as we know, situated in front of the origin of the aorta, so as to conceal it completely—we experienced a sensation that was plainly distinct from the vibratory movement which answered to the contraction of the artery, and which consequently coincided with the dilatation of the ventricles: this vibratory movement was feeble and indistinct during the dilatation of the artery, and the simultaneous contraction of the ventricle.

4. Now what does this vibratory movement indicate? As it was impossible to apply the ear directly on the point corresponding to the perforation in the sternum, I used my fore-finger as a sort of stethoscope; then, putting my ear to one point along the course of my finger, I could distinctly recognise a well-marked clacking sound (*bruit de claquement.*) Several times I, as well as some other gentlemen, repeated this experiment, varying it by applying the ear against the angle formed by the articulation of the second metacarpal joint with the first phalanx of the fore-finger, and always obtained the same result.

5. In vain I sought to hear a double sound; only one could be perceived; and this was sharp and short like that of the second period. Moreover it coincided with the contraction of the artery, and consequently with the shutting of the sigmoid valves thrown back by the column of blood. The interposition of the finger, as an auscultatory medium, had the advantage of combining the perceptions furnished by the sense of touch with those furnished by the sense of hearing.

6. The cause of the second sound was therefore manifestly owing to the vibration or tremulous movement of the sigmoid valves of the aorta and of the pulmonary artery thrown back (refoulés) by the column of blood which tends to flow retrograde at the moment of the contraction of the vessels. This second sound coincided with the dilatation of the ventricles, being short like it, and also with the contraction of the auricles.

7. So much for the second sound; let us now consider the cause of the first one. Believing that this, the first, sound had its origin in the movements of the auriculo-ventricular valves, I placed my finger on every point in the circumference of the basis of the ventricles, in the hope of finding on the level of the tricuspid and mitral valves a tremulous vibration analogous with that which I had felt so distinctly opposite to the seat of the sigmoid valves; but no such movement could I discover. Applying my ear, sometimes directly and sometimes with the aid of my finger (as already described), on every accessible point of the basis of the heart, no sound was ever to be heard. I feel therefore convinced that the auriculo-ventricular valves are completely silent (aphones), as well as every point on the surface of the heart, except immediately over the seat of the sigmoid valves; and consequently that I must seek elsewhere for the cause of the first sound.

8. If the first sound has not its seat in the auriculo-ventricular valves, and if all the other points of the surface of the heart are equally aphonous, and if the sounds which they do communicate to the ear are merely sounds of transmission, may it not be possible that this first sound has the same seat or locality as the second—viz. the sigmoid valves of the aorta and pulmonary artery? and may it not be that the first sound is the result of the replacement (*redressement*) of these last-named valves by the wave of the blood from the ventricles, as the second one is the result of the closing together of these valves by the retrograde movement of the blood. This idea seems to me to be confirmed by the following considerations:—

(*a.*) In the case of the present curious malformation, the maximum of the intensity of the first sound was certainly in the same place as the maximum of the intensity of the second.

(*b.*) The character of the first sound was of exactly the same nature as that of the second one, except only in point of intensity which was less, and of duration which on the contrary was greater.

(*c.*) If the first, as well as the second, sound be seated in the sigmoid valves, it must follow that all diseases of these valves will have the effect of altering more or less these two sounds. Now this occurs constantly. In all the observations which I have collected upon this subject, I find that the two sounds are altered in their tone, acquiring more or less of a blowing or rasping character.*

(*d.*) We need not be surprised that no sound accompanies the movements of the auriculo-ventricular valves, seeing that these valves are not loose, but tied down by tendinous cords attached to their free edges and their ventricular surfaces. Add to this that, the contraction of the ventricles taking place in a slow, and at it were a successive manner, the movement of the apex of the heart towards its basis, and consequently the lifting up of the auriculo-ventricular valves must take place in the same manner, and therefore without any vibration. Besides it is evident that, in cases of thickening of the mitral and tricuspid valves, these valves must become more or less vibratile, and the sound which results from this condition will be confounded with that of the sigmoid valves.

(*e.*) To this explanation it may perhaps be objected: if the seat of the sound is in the sigmoid valves, how is it that the maximum of the first sound is at the apex of the heart, and not at its basis on the level of these valves? True, at the bed-side of a patient, the maximum of the first sound is over the apex of the heart when the ventricles contract with so much vigour, that it strikes strongly against the thoracic parietes; but, when they contract feebly, and the throb therefore against the chest is weak, I am confident that it is behind the sternum, and on the level of the pulmonary and aortic orifices, that the maximum of the first sound will be heard; unquestionably it has been behind the sternum, and sometimes in this place only, that I have been able to perceive the first sound in cases of hydro-pericardium. Let us not forget that the first sound is composed of two phenomena quite distinct from each other—viz. 1, the valvular sound, and 2, the shock of the apex of the heart against the chest; hence it is that, in cases of anæmia, chlorosis, and of certain organic affections of the heart, the first sound is so very sonorous as to acquire a metallic tone, and sometimes to conceal almost entirely the second sound.

*General Conclusions.*—From the preceding observations, it appears to me rational to infer that the two sounds of the heart have their seat at the origin of the pulmonary artery and aorta, and their cause in the clacking of the sigmoid valves; that the first sound, which coincides with the systole of the ventricles and with the dilatation of the arteries, is the result of the elevation or replacement of the sigmoid valves (du redressement des valvules sigmoides, prealablement abaissées); and that the second sound, which coincides with the diastole of the ventricles and with the contraction of the auricles, is the result of the closing together of the same valves folded back by the retrograde wave of blood. On the one hand, the simplicity of this theory, and the ready and natural explanation which it gives of all the facts with which I am acquainted, may be adduced

* M. *Cruveilhier* qualifies this assertion by subsequently adding: "let it be understood that I am now speaking only of those cases in which the mitral and tricuspid valves were perfectly sound."

as a proof of its truth; and, on the other hand, the considerations into which I have entered seem to me to have all the weight of a rigorous and direct demonstration. The circumstance of not being able to perceive the first sound with the finger placed in the manner of a stethoscope around the orifices of the aorta and pulmonary artery is by no means a peremptory argument against the doctrine now proposed; for it is of almost daily occurrence that we find that the ordinary stethoscope does not communicate to the ear either the sounds of the heart or those of respiration, when they are very feeble. In conclusion, may we not assert that the first sound must have its seat at the sigmoid valves from the mere circumstance that its seat is not elsewhere?—*Gazette Medicale.*

---

### M. Dubois on the Auscultatory Signs of Pregnancy.

In our last number, we noticed some of the observations of this accomplished accoucheur on the signs furnished by the state of the mammæ in pregnant women: we shall now pick out a few of the most interesting remarks from his lectures on the other signs.

*Auscultatory Signs.*—The uterine *souffle* is usually perceptible about the fourteenth or fifteenth weeek of pregnancy: the period at which it may be first heard, being, no doubt, dependent upon the amount of development of the uterus and its elevation above the os pubis. The point at which it is most frequently audible is towards the middle of the heighth of the uterus on its anterior or lateral (generally the left side) part. In this respect M. *Dubois* differs from M. *Naegele,* who states that the common situation of the uterine blowing sound is in one of the inguinal regions, extending thence upwards. In most cases, the space over which it may be heard is limited to a circle of two or three inches in circumference. A curious circumstance connected with this sound is the occasional changeableness of its situation; on one day it is inaudible at a spot where it had been distinctly heard the day before, and *vice versâ.*

Obstetrical auscultators should be aware of this fact; else they will be apt to be perplexed in some cases. We may mention likewise that the uterine *souffle* varies much at different times in its loudness and distinctness, being one day scarcely audible, and on the next, perhaps, very distinct.

That the development of this sound is somehow dependent upon the circulation of the blood through the uterus, appears from the fact that it is always much enfeebled, or even altogether suspended, by the contractions of the organ during parturition—a fact which abundantly proves that the sound cannot proceed from the pressure of the gravid uterus on the iliac arteries, as some writers have alleged. The striking resemblance of the uterine *bruit* to that perceived in erectile tumors, and in aneurismal varices, confirms the above opinion. M. *Dubois* objects to the appellation of placentary or utero-placentary being applied to this blowing sound, for the reason that, although its locality most frequently corresponds with the attachment of the placenta, it continues to be audible for some time after the expulsion of this body, and in other cases after the death of the fœtus.

The other sound, that of the fœtal heart, is a still more decisive sign of pregnancy: the number of the pulsations varies, according to the experience of M. *Dubois,* from 135 to 150.* This tictac sound is usually most distinctly perceived on the anterior part of the abdomen somewhat to the left side: it is

---

* It is a curious circumstance, that the late Dr. *Hamilton,* so long the distinguished professor of midwifery in the University of Edinburgh, most stoutly rarely audible before the completion of four, or four and a half, months of pregnancy.

*Changes in the Cervix Uteri.*—It has been asserted by some obstetrical writers, that the diminution in the length of the cervix is gradual and regularly progressive during pregnancy; thus, that by the end of the fourth month, it has lost about one-third of its length; by the end of the fifth month, one-half; by the end of the sixth, three-fourths; and by the end of the eighth, it is not more than about two lines long. It must not, however, be imagined that in all cases this diminution of the neck of the womb is so uniform as to enable the accoucheur to predict the period of pregnancy.

M. *Dubois* mentions a case in which he had an opportunity of examining the uterus in a woman who died in the eighth month, and in whom the cervix uteri was found to be as long as it usually is during the first months; and he cites another instance where a woman stated that she was near the period of her confinement, but her accoucheur, finding that the cervix uteri was not at all shortened, expressed his disbelief that such could be the case; the result however, soon proved the inaccuracy of his opinion.

In many cases, the outline of the distended uterus may be felt through the abdominal parietes: this examination is always best made when the woman is in bed and early in the morning before food is taken: if the bladder and bowels have previously been emptied, the examination will be more easy and satisfactory.

M. *Dubois* cites two or three curious cases, in which the pains of seeming labour came on with all the other usual accompaniments at the time expected by the woman, although it afterwards proved that she was not even pregnant.—*Gazette des Hôpitaux.*

---

### NUMBER OF SUICIDES IN FRANCE IN 1839.

During the course of this year 2,717 cases of self-destruction have been reported by the authorities of the different departments; of this number, 698 were committed by females; and 486 occurred in Paris alone.

We cannot, however, regard this as the entire number of suicides perpetrated throughout France, during the year; for very many undoubted cases, says the reporter, are, either from insufficient data or at the importunity of relatives, registered as deaths from accident. The following table shews that the dreadful crime of self-destrucion has been yearly increasing during the last four years:—

| | |
|---|---|
| 1836 | 2,310 |
| 1837 | 2,413 |
| 1838 | 2,556 |
| 1839 | 2,717 |

(A lamentable picture of human depravity!)—*Gazette Medicale.*

---

maintained that the pulse of the child in utero, and also after birth until breathing commenced, seldom exceeded 60 or 70 pulsations in the minute. In our notice of his last work, in the number of the Medico-Chirurgical Review for July 1836, we questioned the accuracy of the Doctor's assertions, and incurred in consequence his wrathful criticism. Out readers may find it worth their while to revert to his pamphlet, and our rejoinder, in the number of this Review for January 1837.—*Rev.*

### Case of Palsy of both Facial (7th pair) Nerves.

The following very curious and rare case is well deserving of being recorded.

A young lady, 22 years of age, consulted M. *Magendie,* in April 1840, in consequence of the following symptoms which had made their appearance about a fortnight previously. The earliest symptom was a slight embarrassment in the movements of the left eyelid; this was soon accompanied with an inability to contract the left temple and left half of the forehead. The next symptoms were sensation of numbness in the left side of the tongue, but without any impediment in its movements, and an increased sensitiveness of hearing, so that the gentlest sounds caused a painful resonance in the left ear. These two last symptoms did not last above 24 hours; but the paralysis was permanent. At the period of her consulting M. *Magendie,* there was considerable distortion of the features on the right side, especially of the mouth and chin; also an inability to contract the forehead and to close the left eyelids. The left side of the upper lip hung down below the level of the other side, and the left side of the lower one was equally paralysed: the saliva flowed involuntarily from the left angle of the mouth. The left cheek was drawn to the right side, and was stretched and firmly applied to the gums and teeth: it swelled somewhat during the act of expiration, and fell down during that of inspiration. While eating, the food was apt to collect in the left side; and while speaking, laughing, &c. the deformity of the features was always greater.

M. *Magendie* recommended a trial of galvanism with acupuncturation; one needle was to be inserted over the parotid gland and another over the supra-orbital, the infra-orbital, and the mental foramina successively. These needles were then brought in connection with the conductors of *Clarke*'s machine, the wheel of which was at first turned round slowly and afterwards more quickly. Each galvanic shock produced painful dartings over the whole of the affected side of the face; but it was observed that the muscles contracted but feebly. This treatment was ordered to be repeated every day.

For the first five days it seemed to have little effect, and on the sixth, a new set of symptoms made their appearance; the right side of the face now becoming affected in a similar manner. The right eye could with difficulty be closed, and the right side of the forehead could not be moved; the distortion of the left side was at the same time considerably less than it had been before. On one day the patient experienced a numbness in the right half of the tongue, and a painful sensitiveness to sounds in the right ear. M. *Magendie* ordered that both sides of the face should be galvanised daily in the manner that we have explained: the contraction of the muscles under the influence of the shocks was observed to be much less than in health. Little benefit however seemed to be derived from the treatment, and on the 15th of April, (13 days after her first visit to M. *Magendie,*) the following report was made of her case.

There is now no longer any distortion of the features; they are regular, but motionless and impassable, so that they do not express by any change, except in the colour of the face, the emotions of the patient. The eyeballs seem to be unusually large, in consequence of the inability to close the lids; and the hanging down of the eyebrows gives a most unpleasing character to the physiognomy: the tears are constantly flowing down the cheeks. The patient cannot contract any part of the forehead; the alæ of the nose are much flattened, and inclined in towards the septum. The lips have lost all their contractility, so that the speech is much embarrassed, and the pronunciation of labial sounds is impracticable. Mastication is equally difficult; for the food is always getting between the gums and the cheeks, and the patient is obliged to use her fingers to displace it. The cheeks are flat and hang down, so that the face looks lengthened and much older than in health.

From the phenomena now mentioned, it is obvious that the muscles under the influence of the facial nerves of the 7th pairs have lost almost entirely their contractile powers. The general health has however remained perfectly good; the appetite being regular, sleep undisturbed, &c.

After a few days' longer continuance of the galvanism, the face was observed to be somewhat drawn to the left side—a good sign; for it implied that the muscles on this side were recovering their motility. At the same time the patient found that she could move a little the left angle of the mouth, close the left eyelid, and contract somewhat the left side of the forehead. The galvanism being persevered with, the muscles of the right side soon began to contract more forcibly under its influence, and to be also more obedient to the will.

In the first week of May (25th seance), the features had nearly recovered their perfect regularity;—not indeed as in the second stage of the case, when the absence of distortion was owing to a double paralysis, but in consequence of a double cure—which was only slightly disturbed when the patient spoke or laughed. The tears and the saliva were no longer excreted involuntarily; the sides of the nose were not pinched in as before, and the patient could now masticate her food without being obliged to be every now and then putting her finger in her mouth. M. *Magendie* recommended that the needles should now be implanted only into the substance of those muscles whose motility seemed to be not quite so perfectly recovered as that of the others. In the course of a week or two more, the cure was pronounced to be complete in every respect.

In concluding the report of this curious case, it should especially be kept in mind that the sensory twigs of the *portio dura* on either side were not at all affected; the sensibility of the face remaining as perfect as in health throughout the continuance of the muscular paralysis.

It may be objected by some that the continuance of the sensibility unimpaired might be owing rather to the integrity of the fifth pairs, than of the sensory twigs of the seventh pairs. But the following fact, which was repeatedly observed by M. *Magendie* and pointed out by him to several pupils, disproves this idea. If, when a needle was inserted into the parotid gland, it reached directly a twig of the *portio dura,* that moment the pain radiated along all the ramifications of the pricked nerve. The sensory filaments of the nerve were therefore not paralysed as its motory were.

We can readily understand how the seventh pair should have retained its sensibility under such circumstances; seeing that this is supplied or communicated to it by the fifth pair; for, if we divide the *portio dura* between the stylo-mastoid foramen and the anastomosis with the auriculo-temporal branch, the extremity of the nerve which corresponds to the face does not lose its sensibility, although all power of motion in the parts is abolished.* It thus appears that the *portio dura* does not derive its sensibility directly from the encephalon, but indirectly and by the way of anastomosis, beyond the point where we have made the section.

This circumstance explains why palsy of the fifth pair induces a loss of sensibility in the seventh pair also; the sensory filaments of the latter emanating, so to speak, from the former.

In the patient, whose case we have given above, both nerves of the seventh pair were paralysed, while the fifth pair remained quite intact.

*General Considerations on Paralysis of the Seventh Pair.*

This pair of cerebral nerves is much more frequently affected with palsy than the fifth pair; moreover, the disease in the one case is much less serious than when the latter nerves are implicated. In the first case, the motility only

---

* For an account of the experiments which prove this, consult the two volumes of *Magendie,* Leçons sur le Systeme Nerveux.

is impaired or lost; in the second, the general sensibility of the face and the special sensibilities of sight, hearing, smell, and taste, are more or less compromised, with or without a loss of motion in the lower jaw at the same time.

Palsy of the seventh pair is not accompanied with any lesion in the nutrition of the parts which it supplies; while that of the fifth pair is often attended with most serious changes, amounting sometimes to gangrene in different parts.

Palsy of the seventh pair on one side is not unfrequent; but it is exceedingly rare that both nerves are affected at the same time. The preceding case is almost unique: it confirms what experiments had previously taught us, that the division of both nerves causes a complete immobility of the whole of the face.

The most important point to determine in cases of palsy of the *portio dura* is, whether the disease be simply local, or whether it is connected with, and dependent on, disease within the cranium.

Of all diseases of the brain, hæmorrhage is that which is most frequently accompanied with paralysis of the face. If there be palsy of the limbs of the same side, the diagnosis cannot be doubtful. But the latter symptom may be absent, although there is hæmorrhage; as was shewn in the case of the late Baron *Dupuytren*. We must therefore be on our guard not at once to pronounce a case free from danger, because the face alone and not the limbs at the same time are affected with paralysis.

M. *Andral* lays much stress on the following circumstance to guide our diagnosis. In cerebral hæmorrhage the features are much less distorted, and the muscles of the face continue to have some power of motion: *the patients can readily, on the palsied side, close the eyelids and contract the forehead.* On the contrary, when there is lesion of the nerve without cerebral mischief, the distortion of the features is much more considerable, and all muscular contractility is usually gone: *the patient cannot close his eyelids, or contract the forehead.*

When we have reason to believe that the paralysis of the seventh pair is connected with any recent lesion of the brain, we must carefully avoid any stimulating remedies, and galvanism among the number.

But when the paralysis is of local origin, there is no remedy so efficacious as galvanism to excite the dormant powers of the nerve. M. *Magendie* recommends that we should act on the fifth pair as well as on the seventh, in consequence of the influence which the former has on the functions of the latter nerve. For this purpose, one needle should be inserted into the parotid gland, and another immediately over either the supra-orbital, the sub-orbital, or the mental foramen; or, what is better, over each of the foramina in succession. If during the treatment any muscle or muscles are more refractory than the rest, a needle should be inserted into it, and the galvanic stream be sent directly through it. Some patients object to the use of the needles, although the operation is attended with scarcely any pain. When such is the case, we must be satisfied with merely applying the conductors: one of these should terminate in a flat surface, and be applied in front of the ear; and the other in a lengthened globular end which can be drawn along the internal surface of the cheek and lips. In this way the galvanism may be made to act on the palsied nerves; but its action is much less efficacious than when the needles are employed at the same time. The average duration of the cure of paralysis of the *portio dura* by galvanism may be stated at about twenty *seances*. The machine which M. *Magendie* has exclusively used for some years past is the electro-magnetic instrument of *Clarke*. He very rarely prescribes any medicine either for internal or for external use.—*Gazette Medicale.*

*Remark.*—The preceding case we should pronounce to be one of *hysterical paralysis.* The suddenness of the attack, while the general health was and continued to be good, and its transference to the fellow nerve on the opposite side lead us to this conclusion. The comments however, which the author makes, and his account of *Magendie's* treatment of such cases, are highly interesting.—(*Rev.*)

---

### IODURETTED MERCURIAL SYRUP FOR THE TREATMENT OF SECONDARY SYPHILIS.

Dr. *Gibert* has for some time been using the following syrup in many cases of obstinate secondary venereal affections.

Take of

| | |
|---|---|
| Bi—or deuto-ioduret of mercury . . . . | 1 part (gramme) |
| Ioduret of potassium . . . . . . . . | 50 parts |
| Distilled water . , . . . . . . . . . | 50 parts |

Dissolve, filter through paper, and then add of

| | |
|---|---|
| Very clear syrup . . . . . . . . | 2400 parts. |

The usual dose is a table spoonful, which may be said to contain one centigramme of the bi-ioduret of mercury, and 50 centigrammes of the ioduret of potassium. The syrup has no unpleasant taste and possesses the great advantage of remaining unchanged for a great length of time.

The proportion of the ioduret of potassium in the syrup is more than sufficient to hold the mercurial bi-ioduret in solution; but, besides that it serves to counteract the tendency of the latter to decomposition, it is in itself a useful remedy; whence the excess is rather an advantage than otherwise.

M. *Gibert* also occasionally orders the bi-ioduret in the form of pills; thus:—

Take of

| | |
|---|---|
| Bi-ioduret of mercury . . . . . . | 0,10 grammes. |
| Ioduret of potassium . . . . . | 5,00 —— |
| Powdered gumarabic . . . . . | 0,50 —— |
| Honey . . . . . . . . . . . . | q. s. |

Make a mass to be divided into 20 pills, of which two or more may be taken daily.

By means of these formulæ, M. *Gibert* tells us that he has succeeded in curing several cases of inveterate syphilis and scrofulous cutaneous disease, which had long resisted every other remedy.

---

### BARON LARREY: HIS MEMORABLE CAREER.

The great captains of former times have set the example of devoting the leisure of their old age to the recounting, for the benefit of their successors, the history of their campaigns and military labours. Some of the masters of our art too have followed this most useful plan, blending the impressions of their army life with the results of their professional practice, and thus dramatising science by bringing it in contact with the events of the age. The works of *Ambrose Paré*, for example, not only constitute a venerable portion of the records of surgery, but at the same time shed much light on the state of French society at the period; they combine the interest of a chronicle and the historical value of a scientific treatise. Following the example of the father of French surgery, Baron *Larrey* has communicated to the public, in a series of admirable works, the results of an active life, which, next to that of the immortal Huguenot (Duke de Sully), is assuredly one of the most remarkable on record for its duration and its varied interest. Like his great model, he has taken up the pen only to narrate what he has seen and what he has done: a literary proceeding that, for a length of time, seems to have fallen into desuetude. He commenced active service in 1787, in which year he embarked for America as surgeon-major of the Royal Marine, and was constantly engaged down to the battle of Waterloo, where he was severely wounded. From that period to the present time he has not been idle; the volume which he has recently published bears ample testimony to this. Although rather obstinately attached to many of the doctrines and practices of former times, and unwilling to admit the value of some of the modern discoveries, it is impossible to refuse the highest praise to his numerous works, all of which bear the stamp of great practical value. Everywhere we meet with a clear detail of facts in all their points of view, a quick discernment of their real importance, a fair spirit of deduction applied to practice, and a sound judgment based upon the results of an immense experience.

When we think of the grandeur of the scenes on which Baron *Larrey* acted with a zeal which has never been surpassed; when we call to mind how many times the same problems of military surgery were brought under his persevering attention, and remember the multitude and variety of the operations which he has performed himself, or which have been performed under his immediate inspection, it is impossible not to rest with an entire confidence on the recollections of so colossal a practice, and we feel a degree of hesitation in questioning the propriety of any of his opinions and precepts. The further that we are re-removed from that memorable epoch of battles, and victories, and disasters, during which the genius of war converted the whole of Europe into one vast theatre of clinical surgery, the more highly will be estimated the importance of works replete with precepts dictated, so to speak, on the field of battle, like the bulletins of the Emperor himself. Never probably again will so many and so varied striking events be compressed into the career of one life; and never, we trust, will there be so ample a field of instruction in all the numerous departments of military surgery. He who from Egypt to Waterloo has stanched the wounds alike of conqueror and conquered, he who for thirty years has personified in himself the heroism of humanity, and has drawn around him all the distinguished men who have contributed during that time to advance the progress of our art, like rays all verging to or springing from one centre, diffusing among them the light of his own intelligence, cannot but be regarded with singular interest and admiration. It is not in his published works alone that we are to seek for the proofs of his presiding mind; we must compare the state of military surgery at the beginning of our revolutionary war with its present highly-improved condition, and examine the numerous works of those men who have been formed under his guidance, to appreciate fully the mighty good that *Larrey* has done. The same remark is applicable to another bright ornament of modern surgery in France, we mean M. *Dupuytren:* his published works are far from being a complete expression of the striking individuality of his career and of the benefits which he conferred on medical science.

The recently-published volume of Baron *Larrey* closes the series of the voyages and campaigns, which he has so well described. It contains, besides, a new notice of the immoveable apparatus for the treatment of fractures and certain wounds of the soft parts, and sets in a striking point of view the advantages of its adoption in the surgery of actual warfare. There is also a reprint of the interesting memoir on the physical constitution of the Arabs, which was read some time ago at the Institute; the accuracy of the description has been amply borne out by the testimony of our military surgeons in Algiers, &c. and affords a striking proof that the illustrious author could extend his observation beyond the field of professional labour, and that he viewed every object around him with the keen glance of a philosopher.

Since the disaster of Waterloo, *Larrey* has visited Great Britain,—the Pyrenees, where on a pious journey to his native village he had the happiness of meeting his first teacher, a venerable priest now ninety years old: for, like *Broussais*, he was a child of the church,—Italy, which he had once traversed with our victorious armies, and Rome, where he met Napoleon's august mother, who, though blind, at once recognised the voice of one of the legatees of her illustrious son.

Wherever he went, he inspected with the greatest care all the hospitals, medical schools, museums, &c. taking notes of what he saw and heard, and comparing them with the results of his own individual experience. In England, especially, he made himself thoroughly acquainted with all the details of the medical service, and modes of instruction adopted there, and with the various charitable and other institutions of the country: and, on his return to France, he drew up a report on the state of the English barracks and hospitals, which he communicated to the minister of war.

Let us join him in offering our homage to our *confreres* on the other side of the channel for the distinguished reception which he met with every where among them; in all the hospitals he was received as a master, and honoured as one of the glorious heroes of the imperial Iliad—a useful lesson to ourselves, who are too apt to be just only to the dead.

The caustic genius of the French character often prevents the due appreciation of talent in our own country; we seem to be unwilling to leave alone the glories achieved under our own eyes; and, like our ancestors who, in a day of puerile rage, destroyed the statues and other ornaments of our cathedrals, we, to gratify our sarcasm or ingratitude, too frequently attack the bright fame of our cotemporaries. Alas! we must confess, that even some of his illustrious patients, men who owed their lives to his skill and humanity, have too often forgotten their benefactor in the tranquil life of peace. We could mention the name of one general, who was saved by him more than thirty years ago, and who has been all that time trying to learn gratitude, and has not yet succeeded in acquiring it. It would seem that the Emperor had had a presentiment of such conduct, and that, as a recompense to his friend, he decreed by his will immortality to the name of *Larrey.—Gazette Medicale.*

---

### ON THE FORMATION OF AN ARTIFICIAL ANUS: DISCUSSION AT THE ROYAL ACADEMY.

M. *Amussat* stated that he had performed the operation in three different cases, since the date of his former communication to the Academy.*

In the first case the patient was affected with a cancer of the omentum, and the diseased mass was found on dissection to have compressed the colon, where it is about to form its sigmoid flexure; there was complete retention of the fæcal matters. M. *Amussat* established an artificial anus in the ascending portion of the intestine, following the plan recommended by *Callisen*. Having exposed the bowel on its posterior surface, in the right lumbar region, he first passed a thread through it with the view of securing it to the edges of the wound; he then opened it with a trochar and afterwards extended the aperture in a longitudinal direction. A quantity of fæcal matter was at once discharged. Some time afterwards evacuations took place by the natural passage.

In the second case, the patient, sixty years of age, had, from the same cause as in the preceding instance, an obstruction of the bowels which had continued for nearly fifty days; the cancerous affection was seated at the upper part of the rectum. For a length of time nothing had been discharged per anum except liquid matters mixed with blood and mucosity. M. *Amussat* established an opening in the descending portion of the colon, and immediately a quantity of

* Vide Medico-Chirurgical Review, for April, 1840, p. 539.

fæculent matter escaped from the wound. The bowels remained inactive; and, although no inflammatory re-action followed, the patient died on the 10th day after the operation, from the progress of the cancerous disease.

The third case occurred in a man who had been in excellent health, until suddenly seized with an obstruction of the bowels. The cause could not be discovered; and it was almost impossible to say whether the obstruction was situated in the large or in the small intestines. In this case, the operation of *Littre* was performed, although M. *Amussat* is not favourable to it upon the whole, but gives the preference to that recommended by *Callisen*. By opening the cæcum on its anterior surface, in the present case, he thought that there was a better chance of discovering the seat of the obstruction, whether it was in this bowel, or whether it was higher up. Having exposed the cæcum, he first secured it in its place by means of a thread passed through it, and then made an opening into it. The patient, who was in a state of extreme prostration, died in the course of twenty-four hours after the operation. On dissection, it was found that the obstruction was situated about the point of union of the transverse and descending portions of the colon. It had probably been induced by the irritation caused by a chicken-bone which was found lodged there.

M. *Amussat* stated that the patient, on whom he had operated after *Callisen*'s plan two years ago, remained in perfect health.

. . . . . . . M. *Gimelle,* one of the surgeons of the Hôtel des Invalides, considered that M. *Amussat* had rendered an important service to surgery by restoring the operation of making an opening into the intestine without wounding the peritonæum; but he could not understand his motives for adopting the plan of *Littre* in the third case, where it is admitted that the seat of the constriction could not be determined before the operation,

It is to be remembered that *Littre* proposed his method only under more favourable circumstances; viz. when the distended bowel projected outwardly, and thus presented itself directly, as it were, to the scalpel.

. . . . . . . . M. *Amussat* said that the patient in question, in whose case he had been condemned for operating, was able to retain three enemata. It was therefore, he thought, presumable that the obstruction was situated high up; and, as it was impossible to discover its exact position, he was naturally led to prefer the plan which enabled him to reach as high a portion of the gut as possible. The difficult point for the surgeon to determine is how he should act under such circumstances; if nothing is done, the patient is almost inevitably lost; and if an operation is attempted, he (the surgeon) is exposed to the reproach of having hastened the fatal termination. We have seen that one of my patients, whose death appeared to be imminent, has been saved, and still lives to bear testimony to the success of the operation.

. . . . . . . . M. *Velpeau* :—In canvassing the difficult question at present before the Academy, it should be borne in mind that there is a very marked difference in different cases as to the length of time during which an obstruction of the bowels may continue with impunity: this holds good both in disease and in health. One thing has especially struck me in listening to the observations of M. *Amussat;* some of his patients have had alvine evacuations by the natural passage a few days after the performance of the operation. Now how should this be, if there was an actual and veritable obstruction? And, if such an obstruction did not exist, why operate at all? We have been told that one of his patients has survived for two years, and is still in good health; and yet this patient was pronounced to have scirrhus of the intestine. In what way are we to understand that the operation has been the means of prolonging his life? and how are we to imagine that the establishment of an artificial anus should be a cure for cancer? M. *Amussat* attributes the constriction of the bowel in his third case to the presence of a small bone found in the obstructed part; but may we not suppose that the stricture was of anterior date, and was rather the cause than the effect of the lodgment of the bone? The difficulty of forming an exact diagnosis as to the seat of the disease is extreme, and, in spite of the interesting observations now read to us, is still quite as great in my opinion as ever. When should we operate? This is the embarrassing question; at least in cases of cancerous affections of the bowels. As to the obstruction of the bowels from other causes, as for example in the last case narrated by M. *Amussat,* the operation is certainly indicated on principle; but then how are we to determine the exact seat of the lesion? And, setting apart those cases where we can determine this, I do not believe that the operation of *Callisen,* so much recommended by M. *Amussat,* can ever be generally applicable.

M. *Amussat* replied to the objections adduced by his honourable colleague. As to the circumstance of the bowels relieving themselves by the natural passage after the formation of an artificial anus, it deserves to be mentioned that this occurred not only in my cases, but also in those reported by *Littre.* The explanation is probably this: the cancerous mass becomes more and more softened until ultimately it is detached, and then the passage which it obstructed becomes comparatively free. His doubts as to the real nature of the lesion in the case of the patient who still survives, appear to me unreasonable, as my diagnosis at the time was confirmed by MM. *Recamier, Foville,* and *Breschet.* The slow prognosis of the disease may be accounted for by supposing that there is an incarcerated cancer of the parietes of the rectum.(?)

M. *Guersent* mentioned two cases of obliteration of the intestines: the first occurred in the famous dancer Goyon, who had been long afflicted with a tumor in the rectum. He died in a state of extreme emaciation in the Maison de Santé; and after death it was found that the gut was completely obstructed by a cancerous ring or constriction. In the second case, symptoms of strangulation came on quite suddenly, and no external tumor could be detected. On dissection, a cervical vertebra of a fowl was found placed across the intestine, so as quite to interrupt the passage of the feculent matter.

........ M. *Lagneau.*—It has been said that the re-establishment of the alvine evacuations by the natural passage, after the performance of the operation, cannot be easily accounted for, if the obstruction had been a veritable one. But let us consider that, after the discharge of the fæces by the artificial opening, the irritation which their presence caused gradually subsides, and, the parietes of the intestine becoming freed from their previous engorgement, the passage is thus restored for the transmission of their contents.

........ M. *Cornac* expressed his regret at not having been present at the reading of M. *Amussat's* paper. He had travelled with the patient on whom the operation had been successfully performed two years ago, and could bear ample testimony to the health which he now enjoyed. The patient, in his joy at having been saved by the operation, has often said that if *Talma* and *Broussais* had been treated in a like manner, they might perhaps have been living at the present time.

........ M. *Breschet.*—I perfectly agree with the preceding speaker in the view which he takes of the operation and of its results, and most willingly give the credit due to M. *Amussat* for the happy modification of it which he has introduced. But I cannot agree with him in his regret that it was not performed on *Talma* and *Broussais.* I saw both those illustrious men in their last illness, and do not hesitate to affirm that neither was in a condition for such an operaaon to be attempted. *Talma* was visited by *Dupuytren,* who completely coincided in this opinion and expressly stated, that in his judgment there was nothing that could be done; for, besides the disease of the bowels, there existed an aneurism of the heart, which would in all probability have speedily carried him off, perhaps suddenly, and on the stage, in one of those parts in which he used to display so much pathos, that of Orestes for example.

After a few words from M. *Cornac,* disclaiming all imputations on any of the medical attendants of those distinguished individuals, M. *Begin* rose to express his dissent from the opinions of M. *Breschet.* Far from thinking, said he, that the disease of *Talma* did not admit of the establishment of an artificial anus, it was on visiting him that I conceived the first idea of it. In my opinion his case was eminently favourable to the success of such an operation, as there was no cancerous degeneration of the bowels, but only a simple constriction at the upper part of the rectum. I am aware that there existed at the same time an aneurism of the heart; but such a complication was not in itself sufficient to forbid making an attempt to relieve the local disease; and I had formed the project, which I communicated to *Dupuytren,* to pierce the pouch in which the fæcal matters were lodged, and to open in the rectum an artificial anus by means of this puncture.

M. *Amussat* expressed his concurrence in these sentiments of M. *Begin.* The case of *Talma,* although he had not had an opportunity of seeing him during life, had always seemed to him to have been favourable to such an operation as he has proposed. Not only was the disease of the bowels not of a cancerous character, but Nature herself had commenced a process of self-relief, there being found on dissection an ulceration on the walls of the constricted portion, or rather on the septum which separated the pouch containing the fæcal matters from the lower part of the gut.

As to the case of *Broussais,* I saw him, continued M. *Amussat,* in his last moments, and can state confidently that there was an almost complete stoppage of the bowels: at the time of his death there had been no discharge for 21 days. The operation was therefore in my opinion quite admissible, and indeed it was on the occasion of my visiting this illustrious patient that I first thought of the plan of my method. I did not dare however to make the first attempt on so distinguished a person.

M. *Breschet,* in opposition to what had just been said of the case of *Broussais,* stated that, so far from there being a complete obstruction of the bowels, there were from time to time considerable evacuations, *debacles,* which the patient himself predicted would take place at particular times. It is easy *now* to say that the operation was indicated, and to form a diagnosis of the case *a posteriori,* after the dissection has revealed the nature of the existing lesion; but circumstances were very different during the life of the patient, and it will always be a most embarrassing question to decide as to the propriety of attempting an operation, when the diseased part cannot be reached with the finger.

M. *Gerdy.*—The operation which M. *Begin* proposed in the case of *Talma* appears to me to be very rational, and I have several times performed it with success on infants with imperforate anus, whether the gut was deficient for some extent, or was nearly closed at its orifice. By leaving a canula in the artificial canal, we may avoid all risk of extravasation taking place. In the case of adults affected with an obstruction of the bowels, it is to be remembered, as already alluded to by M. *Velpeau,* that it may exist for a great length of time in some persons without being attended with dangerous consequences. We have all heard of a surgeon of Rochefort, who, during a voyage from France to Senegal and back again to France, had not one alvine evacuation, until he returned home. He lived for a length of time with this unusual condition of the bowels in very good health.

M. *Begin* stated that the chance of extravasation was much greater in such a case as that of *Talma,* than it could possibly be in cases of imperforate anus or rectum occurring in new-born infants. In *Talma's* case the serous coat of the distended pouch and that of the lower part of the gut were opposed to each other, and hence any instrument passed through the septum must necessarily have penetrated the peritoneum.

### M. CIVIALE ON NEURALGIA OF THE NECK OF THE BLADDER, &c.

This is an affection which is not unfrequently idiopathic and unconnected with any other diseases of the urinary organs, according to the experience of M. *Civiale*. It occurs in both sexes, but is much more frequent in the male; it is occasionally observed in young persons, but generally in adults and in those of advanced years. It may be induced by a variety of causes; either general, such as emotions of the mind, excesses in venery, or in the use of spirituous drinks; or local, such as retaining the urine too long, various lesions of the rectum or womb, a calculus in the bladder, constipation, &c. It is usually accompanied with frequent calls to pass water; with an uneasiness rather than an actual pain in doing so, and this extending to the pubis, epigastrium, and umbilicus, or to the loins and down the thighs; and with more or less anxiety of mind in almost all cases. It may be either single and exist by itself; or it may be complicated with contraction of the urethra, swelling of the prostate, calculus or catarrh of the bladder, &c. The catarrh of the bladder is often secondary to the neuralgic affection of its cervix, according to the experience of M. *Civiale*. One of the least variable of all the symptoms is unquestionably the intermittent character of the vesical distress. It seems, however, to us, that our author is disposed to regard neuralgia of the bladder as more frequently idiopathic and unconnected with any other existing disease of the urinary organs, than most surgeons will admit. He acknowledges himself that in those cases in which the affection is complicated with stricture of the urethra, it will generally cease spontaneously when the disease is successfully treated. We know, too, that, in spite of the great improvements which have been effected of late years in the diagnosis of the diseases of the prostate and of the cervix vesicæ, these are sometimes so obscure in their early stages that they may be readily overlooked. We state this, not with the view of questioning the accuracy of the data furnished by M. *Civiale*, but only to account for the repugnance which some may feel to admit as a distinct entity a morbid condition, which has generally been attributed to various organic lesions either of the bladder itself, or of the contiguous organs. Indeed we are willing to give great praise to him for having drawn the attention of medical men, in so able a manner, to an affection of the urinary passages, which has been too much overlooked, and which according to his experience may exist quite independently of any organic lesion. The treatment, recommended by M. *Civiale*, consists in the introduction daily or every second day of a wax bougie into the bladder, to be left in for from three to ten minutes at a time; in the use of injections of cold or of tepid water into the bladder; the use of douche baths; the external application of the tartar-emetic ointment to the pubic region; the regulation of the bowels, the use of a mild diet, and the avoidance of stimulating drinks.

### *Diseases of the Vesiculæ Seminales, &c.*

These organs, with the excretory ducts leading from them, are unquestionably subject to various morbid states, all of which usually take their origin in a sub-acute inflammation of their structures. M. *Civiale* describes three stages or degrees of this disease; but it is to be remembered that there is always considerable ambiguity in the symptoms.

In the first stage, the erections of the penis are more or less imperfect, the emission of the semen hurried and precipitate, and the sensation which accompanies the act, sharp or even painful. Under such circumstances the daily use of warm hip-baths, and mild enemata, and the occasional introduction of soft bougies along the urethra, may be resorted to with advantage. But the most important means of cure is unquestionably abstinence from sexual connexion; and unfortunately it is the very one which is least likely to be followed by the patient. To persuade a man in the vigour of age, and in whom, in consequence of the disease, the passion is more than ordinarily strong, that his desires are owing, not to an exuberance of vitality, but to a local irritation of his generative organs, is no easy thing.

Perhaps the best mode of effecting our object, is to direct the patient's attention to the effects which are usually the result of sexual indulgence in his own case; and he will confess that, after coition, he almost always experiences a prolonged exhaustion of strength, an unusual tendency to drowsiness, lassitude and sense of cold over the whole body.

In the second stage, the continued discharge from the urethra, when it does exist, becomes more abundant, coition is impossible, the semen escapes during the relaxation of the penis, and it is thin and inodorous. The use of cold applications to the pubis and perineum, of sulphureous douche-baths, of regular bodily exercise in the open air, of mild aperient enemata, and of some of the mineral tonics, constitutes the best treatment in such cases. M. *Lallemand* strongly recommends the occasional application of caustic to the deep-seated portion of the urethra: this, no doubt, is a valuable remedy; but it must be employed with caution.

In the third stage, all sexual desire is entirely abolished, and the erection of the penis is impossible; and the general health is almost always so much impaired, that local treatment holds out no prospect of doing any good. It is by fortifying the strength of the constitution, by travelling much in the open air, by a nutritious but mild regimen, and by living entirely *en garçon*, that any benefit may be expected to be derived. M. *Civiale* has given many valuable instructions both to patients and to medical men, clothed, too, in language altogether worthy of a delicate-minded gentleman, on the general management of cases of this description.

He devotes a chapter to the description of the various sorts of bridles (brides) or obstructions which are apt to be formed in and around the neck of the bladder, and which have of late years attracted a good deal of notice from pathologists. Some of them are membranous, others muscular, and a third set fungous. They arise either from inflammation of the vesical mucous membrane, or from swelling of the prostate gland. Whatever be their form or consistence, they are always situated on the lower edge of the vesical orifice of the urethra. When the obstruction is inconsiderable, the less that is done locally the better. If, however, the evacution of the urine is impeded, or threatens to be so, the steady and persevering use of soft bougies affords by far the best means of effecting a cure.—*Traité Pratique sur les Maladies des Organes Genito-urinaires.*

---

### Section of the Muscles of the Eye in Amaurosis, &c.

M. *Petrequin*, chief surgeon of the Hôtel Dieu at Lyons, recently communicated the following observations to the Academy of Sciences.

"From what I had observed in several cases of squinting, I was led to suspect that amaurosis is occasionally attributable to a spasmodic state of one or more of the muscles of the eye-ball.

In the majority of persons who squint, there is at the same time a greater or less weakness of vision, especially on that side where the morbid deviation is most conspicuous. Now I have found that this visual asthenia, whether this be a primary or a consecutive affection, generally subsides after the section of one or more of the muscles, especially when the patient has recourse to orthopthalmic gymnastics. (What a phrase!) Hence the operation becomes an heroic remedy for amaurosis when it arises from this cause. The following cases will illustrate this position.

*Case* 1.—A young man was admitted into the hospital at Lyons for a white-swelling of one of his fingers. He was also affected with an incomplete amaurosis of the left eye, which I could not trace to any of the ordinary causes. I remarked, however, that in certain movements of the eye there was always a tendency to a deviation inwards. Having repeated my observations during a month, I came to the conclusion that the spasm of the muscles exercised a great influence on the function of vision, and I determined to try the effect of dividing the two internal recti muscles. The sight of the left eye was immediately improved, and five weeks afterwards it was in every respect equal to that of the right one.

*Case* 2.—A youth, 17 years of age, was admitted with an injury of the middle finger which required to be amputated. The left eye had been for a long time almost completely amaurotic, so that he could not count my fingers held up before him; the blindness had come on without any appreciable cause.

In this case too I remarked that in certain movements of the eyes there was a tendency to them being turned inwards; and, acting on the principles which guided me in the treatment on the former occasion, I divided the two internal recti muscles. The amendment was instantaneous; and, on his leaving the hospital three weeks afterwards, the sight was so much improved that he could tell the names of large letters, and distinguish persons at a hundred paces' distance.

M. *Petrequin* states that he has met with equal success in a number of similar cases, since these two occurred.

He has also been led by several observations to conclude that *entropion*, in certain cases, arises from a spasmodic state of the *orbicularis* muscle of the affected eye, and that this species of the disease may be cured by the subcutaneous division of it, as recommended by MM. *Cunier* and *Phillips*. He gives the following case in which he performed the operation.

*Case.*—A man, 45 years of age, came to me with a complete entropion of the right eye; the lower lid was folded and rolled inwards, being curled upon itself in a concentric manner, precisely in the direction of the circular fibres of the palpebral muscle; when the finger was applied upon the part, the contraction was very sensible, especially if a strong light fell upon the eye: the spasm was permanent. Stretching the lower eyelid at its outer angle with a pair of forceps, I passed a narrow bistoury about the middle part of the orbit, on the level of the osseous edge of the socket; then with a see-saw movement (par un mouvement de bascule) I pushed the point of the instrument as far as the free edge of the lid behind the orbicularis muscle, and, in the act of withdrawing it, I divided its fibres completely. The lid becoming immediately ecchymosed—a firm compression was ordered to be kept up upon it. (The result of the operation is not stated.)

M. *Petrequin* closes his observations with mentioning that, in certain cases of blindness arising from opacities and other lesions of the cornea, in which the operation of making an artificial pupil is usually recommended, very decided benefit to vision may be obtained by dividing one of the muscles of the eye-ball, and thus inducing a certain degree of squinting, so that a transparent portion of the cornea may be brought into the axis of the organ. M. *Cunier* has the merit of having first suggested this new method of treatment; and M. *Petrequin* has adopted it in one case, where he divided the superior rectus muscle with marked advantage.

### Division of the Muscles of the Eye in certain Cases of Blindness.

When the central portion of the cornea has become so opaque as to prevent the transmission of the rays of light through the pupil, remaining sound, we may displace the axis of the eye, by dividing one or more of its muscles, so as to induce a certain degree of squinting, and thus bring a transparent part of the cornea in the direct line of vision. Such an operation is much more simple, and greatly less hazardous, than any of the modes which have been proposed to form an artificial pupil.

M. *Florent Cunier,* a well known ophthalmic surgeon in Belgium, claims the merit of having first performed it.

A man, 25 years of age, presented himself on the 21st of June, at his ophthalmic institution, with a strabismus of the left eye, which had existed from infancy. When two years old, he had suffered from a purulent ophthalmia, which had caused the destruction of this eye, and had left on the cornea of the other a dense opacity, which covered nearly the outer two-thirds of its surface; the inner third, which remained transparent, was almost quite concealed in the angle of the orbit, and was visible at those times only when the squint was made to cease. When this was done he could see near objects, by carrying them towards the nose, and then turning the eye as forcibly as possible outwardly. The anterior chamber of the cornea was normal, and the pupil was quite free and readily contracted on exposure to light.

On the 30th of June I divided, says M. *Cunier,* the internal rectus muscle; and immediately the pupil occupied the centre of the orbit, and the squint vanished. The eye being, however, not sufficiently drawn outwardly to enable the patient to see objects conveniently, I denuded the sclerotic as far as the attachments of the superior and inferior recti, but without causing the slightest degree of squinting outwards. Founding my practice on the experiments made by myself and Mr. *Duffin,* I divided the inferior oblique muscle; this was no sooner done, than at once the eye was drawn outwards and somewhat upwards. The ecchymosed blood in the cutaneous wound was rapidly absorbed; but the healing of the conjunctival wound was rather tedious, in consequence of the very considerable retraction of the muco-serous membrane induced by the displacement of the eye-ball. Immediately after the operation, the patient was able to guide himself through my garden; and six days afterwards he came alone to my house.

He now sees so well that he can distinguish the smallest objects, when they are brought near his eye. What is remarkable is, that the pupil has become displaced in such a manner that it is now immediately opposite to the transparent portion of the cornea.

*Case* 2.—A middle-aged man, who had been blind for 12 years, was my second patient. The left eye was completely wasted; and only a part of the outer half of the right one remained transparent. By compressing the left eye, and turning the right one forcibly inwards, he could perceive the form of large objects. I divided the external rectus muscle, and denuded the sclerotic as far as the insertion of the superior and inferior recti; the eye was immediately drawn inwards so as to cause a squint in that direction, and I was gratified to find that the patient was able to see much more distinctly. Eight days afterwards, he could spread out in the market-place his wicker-baskets, which he made to earn a subsistence.

In a third case, in which the operation for forming an artificial pupil had previously been attempted, but without success, M. *Cunier* divided the external rectus; and, although the case was certainly a very unfavourable one, considerable improvement of the vision was effected.—*Gazette Medicale.*

*Remarks.*—This application of ocular myotomy is certainly one of the most ingenious and scientific that has been proposed. The operation for making an artificial pupil being always hazardous, and seldom successful, surgeons will gladly avail themselves of the practice suggested by their Belgian *confrere.—(Rev.)*

---

### ON THE OPERATIONS FOR THE CURE OF STAMMERING.

A well written article, in which the reader will find an accurate description of the various operations which have been recommended and practised for the relief of stammering, appears in one of the late numbers of the Archives de la Medecine Belge (an exceedingly well-conducted periodical, published at Brussels), from the pen of Dr. *Zoude.* We have no intention of re-producing its contents, however valuable they are as forming an historical document, and shall merely give the sensible conclusions with which the writer closes it: they are the following:—

1. Glossotomy is far from effecting a cure of stammering in all cases.
2. When stammering is owing to a disturbed innervation of the tongue, or when it depends upon a shortening of the muscular substance of the *hyo-glossi,* or upon other analogous lesions, myotomy is inapplicable; because, in such cases, it would be necessary to divide the entire thickness of the tongue—an operation which is always highly dangerous.
3. A surgical operation is admissible only when the stammering depends upon a shortening of the *genio-glossi* muscles—as certainly is the case in a great number of instances.
4. In my opinion, the subcutaneous method of dividing the *genio-glossi* close to their attachment to the jaw, without wounding the mucous lining of the mouth, is preferable to any other.

The following judicious remarks are appended to the memoir of Dr. *Zoude,* by the committee appointed by the Society of Medicine at Brussels to report upon it.

. . . . . . . "We have read the work of the honourable member with much interest, and recommend that it should be printed in our Archives as containing a fair account of all that has been written on this operation, (which, by-the-bye, has made much more noise than done good,) and as shewing most satisfactorily that the section of the muscles of the tongue is not always so innocuous as its advocates wish us to believe. We sincerely trust that the attentive perusal of Dr. *Zoude*'s memoir will abate the zeal of those who have been so enthusiastic in the praise of myotomy, by the vain eclat which has been given to the operation, and that, at the same time, it will diminish the willingness of patients to submit to it. We are deeply convinced that, as to the pretended improvements of modern surgery, it is most necessary to guard ourselves against admitting them too readily, and not to lose sight of the truth that the insatiable desire for notoriety and temporary success often makes a man work almost entirely for his own profit, when he is pretending that all that he does is for the progress of science: *Nisi utile est quod agimus, vana est gloria nostra.*"

We (*Rev.*) need not say how entirely we coincide in the justness of these observations. The *doings* of not a few medical men, in this country as well as on the continent, in reference to the treatment of squinting, stammering, &c. by surgical operations, have been any thing but creditable to the profession.

How is it, we would ask, that we have heard so little for the last six months of dividing the muscles of the eye for strabismus, while during the preceding half year every hebdomadal journal was teeming with *fournées* of marvellous cures? Is it that all squinting persons are already cured, and the catalogue exhausted? or is it that the relief produced by the operation has proved only temporary, or that the eye-ball has suffered in some other way from the effects of it?

As to the surgical experiments—for we can call them nothing else—for the cure of stammering, we have from the very first regarded them with suspicion, if not with utter distrust. Some of them, especially those proposed by *Dieffenbach*, and adopted by others, are positively brutal. The very idea of dividing the root of the tongue fairly across with a deep incision through its entire substance, and this, too, for an affection which is generally a nervous one, is worthy only of a butcher, whose province it is not to heal but to mangle.

By-the-bye, Dr. *Zoude* omits to mention, among the various plans of operating, that which has been so loudly praised by a resident in this city, Mr. *Yearsley*, and followed, if we could give credit to his statements, with such extraordinary success. We live in an age of discoveries truly; and certainly one of the most unlooked for is the almost instantaneous cure of stammering by clipping off a small bit of the tonsils or uvula! We read this gentleman's book on deafness, and do not hesitate to pronounce it a thorough *puff*. The materials are put together with a good deal of tact, so as to escape the imputation of quackery; but it is too obvious throughout, that it was got up as a mere means of public advertisement. It has, however, probably served the author's purpose; and he no doubt has had his reward. As conservators, however, of professional character, we feel it our duty to discountenance all such practices; as they only serve, in the long run, to bring contempt upon an art, which when followed with truth, kindness and skill, is, in the words of *Cicero*, almost godlike.—*(Rev.)*

---

### Animal Magnetism prohibited by the Vatican.

This marvel-working science is beginning to attract the notice of the heads of the Papal Church. We suppose that these gentlemen, who have the *cure* of souls, are somewhat jealous of any layman trespassing upon their peculiar province, that of working on the credulity of the mass.

Little would it surprise us to find that animal magnetism, although now forbidden to be practised by the uninitiated, will, at some future time, be taken up by the priests themselves, as a means of exciting the wonder and thus enhancing the zeal of their followers, while at the same time it magnifies their own authority.

However this may be, it has seemed right to the venerable conclave to pronounce against the lawfulness of the act in the present day.

The Bishop of Lausanne and Geneva, during the course of last May, addressed from his palace at Friburg a letter to the sacred penitentiary at Rome, in which, after mentioning all the alleged marvels that have been wrought by animal magnetism—such as somnambulism or ecstatic sleep, clair-voyance or the power not only of seeing when the eyes are closed, but also of predicting events and other similar wonders—closes with these respectful interrogatories:—

"Perceiving strong reasons for doubting that such effects, produced by an occasional and manifestly a disproportionate cause, can be purely natural, I very urgently request your Excellencies to be pleased in your wisdom to decide, for the glory of God, and the advantage of souls redeemed by Christ, whether, supposing that the announced facts are really true, a confessor or a curate may permit any of his penitents or parishioners—

1. To exercise animal magnetism as if it were an auxiliary or supplementary part of medicine;

2. To consent to be plunged into the state of magnetic somnambulism;

3. To consult either for themselves, or for others, persons thrown into this state of somnambulism;

4. To do any one of those three things; even with the precaution of having previously renounced, in the most formal manner, all compact with the Evil One, and all satanic interventions; seeing that, notwithstanding this precaution, some persons have obtained from the employment of animal magnetism all, or at least some, of the effects which have been described."

To this petition, the following pithy answer in learned Latin was sent from the Vatican:—

"Responsum."

"Sacra Pænitentiaria mature perpensis expositis respondendum censet prout respondet:—usum magnetismi, prout in casu exponitur, non licere.

Datum Romæ in S. Pænitentiaria, die 1, Julii 1841.

C. Card. Castracane, M.P.
Ph. Pomella, S. P. Secretarius."

We may avail ourselves of this opportunity to recommend to the perusal of those, who may wish to read a fair and enlightened history of animal magnetism, the work recently published by Drs. *Burdin* and *Dubois*, Members of the Academy of Medicine in Paris, and entitled "Histoire Academique du Magnetisme Animal."

In a well written introductory chapter, the authors endeavour to establish a connexion between all the leading juggleries which from one age to another have made their appearance in the world. They carry the reader, without any forced transition, from the oracles of antiquity to the witchcraft of the middle ages, from the devotees of Loudun to the tremblers of Cevennes, from the convulsionists of St. Medard to the exorcisms of Gassner, and lastly to Mesmerism, which the true believers point to us as the era of the doctrine of animal magnetism.

In the work of MM. *Burdin* and *Dubois* will be found a most faithful history of all the academic discussions which have taken place on this strange subject. Judicious narrators, they class the various facts according to their epochs, so that the reader follows with interest the course—sometimes slow, at other times most precipitate—of this doctrine; and can readily detect the changes and modifications which some of its modern proselytes have endeavoured to introduce.

They have given accurate accounts of the numerous reports which at different times have been made to different academies by their committees, and minute details of the various experiments which have been performed before them.

Among the reports they distinguish with great care those which, after being elaborately discussed by the members of the learned bodies to which they were communicated, may be regarded as the expression of their opinion, from that one which was read before the Academy of Medicine in February, 1826, but was neither discussed nor approved of, and the responsibility of which belongs exclusively to its author, M. *Husson*. This is the famous report which, rejected by the disapproving silence of the learned assembly, was received with so much enthusiasm by the admirers of animal magnetism, and which these gentlemen are so proud of putting forward in the way of preface to the history of their wonderful revelations, as a sort of antidote to the famous report of 1784, signed by the great names of *Franklin*, *Bailly*, *Guillotin*, *Lavoisier*, &c.

M. *Husson*'s report is rather roughly handled by MM. *Burdin* and *Dubois*; and, although they have not maintained all the decorum of academicians in some of their criticisms, it must be acknowledged that the singular position, in which the author has placed himself in reference to the modern occult science, necessarily exposed him to the sharp attacks of reviewers.—*Bulletin Med. Belge.*

---

### On the Medicinal Properties of Fish-liver Oil.

The fish, from the livers of which the oil that has at different times been much recommended in a variety of diseases is obtained, are the cod and one or two species of the ray—*oleum jecoris aselli vel rajæ.* We learn from M. *Tiedemann*, a merchant of Bremen who has long dealt in the article, that there are four kinds of cod-oil in commerce. The livers are packed in a cask, end upwards, and exposed for a length of time to the heat of the sun. When the upper layers are removed, the clearest oil is obtained; this is found to become stronger and darker towards the lower part of the cask. The darkest and thickest is used in the manufacture of chamois leather. MM. *Gouzée* and *Gmelin* state in their memoir (Bulletin Med. Belge, Janvier 1838), that the clearest oil should be used for internal administration; but MM. *Trousseau* and *Pidoux*, in their treatise on Materia Medica, recommend not only the second degree, which has a fishy taste and causes an acrid sensation in the back of the throat when swallowed, but also the third degree, obtained by boiling the residue, and which is of a brown colour, has a most disagreeable empyreumatic smell, and is still more acrid. The opinion of these gentlemen has been confirmed by the experience of most physicians in Germany and Belgium—that the thick acrid oil is much more efficacious than that which is transparent and milder.

Within the last five years, chemical analysis has detected the presence of a small proportion of iodine in fish-liver oil; but it is very doubtful that its medicinal virtues depend upon this principle. However this may be, it is certainly true that the browner the oil is, the more iodine is usually present in it.

That fish oil has been long known as a remedial agent, is proved by the narrative in the Apocryphal book of the Bible of *Tobias* and the Angel: *Tobias* is ordered to take the heart, liver, and gall of the fish that he has caught on the banks of the Tigris, and use them in a prescribed manner. *Pliny* too, in his Natural History, expressly states: "lichenes et lepras tollit adeps vituli marini, murœnarum cinis cum mellis obolis ternis, jecur pastinacæ (raiæ) in oleo decoctum...... Quidam delphini jecur in fictili torrent, donec pinguetudo similis oleo fluat, ac perungunt." The inhabitants in many parts of England, Holland, and Germany have been from time immemorial in the habit of employing cod-liver oil in the treatment of chronic rheumatism, rachitis, &c.; but it was not until *Michaelis* about the middle of last century, and subsequently Dr. Percival (1790) and Dr. Darbey (London Medical Gazette, vol. 3, p. 392), drew the public attention to its effects, that it has been at all generally known to medical men. There are numerous reports of its efficacy in the German Journals, references to all of which are given by Dr. *Delcour* in the number of the Archives de la Med. Belge, for last June.

The physiological effect of fish-liver oil appears to be that of a general stimulant of the whole body. It is apt to produce nausea and even vomiting at first; but after a short time it ceases to do so, and often the appetite very sensibly improves under its use. The urinary secretion is generally increased in quantity, and often a copious deposit of lateritious sediment takes place. The skin and also the uterus are stimulated by its use; hence its utility in several cutaneous diseases and in amenorrhœa. From its established efficacy in scrofulous and other cachectic states which indicate an unhealthy state of the nutritive process, it has been regarded by many as a general roborant, more especially of the alimentary canal and of the lacteal and vascular systems. In most cases where the blood is poor and thin, the best authorities assure us that a long-continued use of fish-liver oil exercises a very salutary influence. If this be the case, we can readily understand how it may act in the cure of scrofula, rickets, chronic rheumatism, inveterate affections of the skin, many diseases of the bones, glands, &c. We have already said that in our opinion the therapeutic properties of cod-oil cannot be justly attributed to the small portion of iodine which exists in it. We are quite aware that in many parts of Holland it is given for a length of time to children of a scrofulous family, to prevent the development of the constitutional affection, so common in that country.

But there is this marked difference between the two remedies; iodine, which is so powerful a remedy against enlargements of the thyroid and other glands, has very little effect in diseases of the bones, whereas it is in these intractable cases that the oil has been found to produce its most beneficial consequences. We are far from denying that the oil may owe part of its properties to the iodine contained in it, and that its action in simple scrofula and in amenorrhœa may depend upon this active principle; but how shall we thus explain its influence in rachitis?

We must here notice the opinion of M. *Hoebeke*, that a long use of fish-oil has in numerous cases been followed by a deformity of the pelvis in women, who had previously borne children without any unusual difficulty. In all his cases the deformity was observed to have affected chiefly the transverse diameter and the outlet of the pelvis—whereas that arising from rickets is usually at the inlet, and in an antero-posterior direction.

Now, although we do not dispute the accuracy of the statements of M. *Hoebeke* and others, we feel much inclined to question the explanation which they give, when they attribute the deformity of the pelvis in women, who had been previously well made, to their having taken large or long-continued doses of cod-liver oil. It seems to us more probable to suppose that the softening of the pelvic bones had been an accidental occurrence, quite unconnected with the use of this medicine. We know from the history of other cases that this disease of the osseous system often commences after delivery and not unfrequently during gestation; that its progress is always increased by the latter state; that it has many points of resemblance with chronic rheumatism; that it usually commences with pains in the back and pelvic region; and that it induces a deformation of the pelvis, which is different from that caused by rickets and similar to that observed by M. *Hoebeke*. The committee, appointed to report upon the memoir of this gentleman, very justly observed that in the district where his cases occurred, the climate was damp and unwholesome, and the inhabitants living in great destitution and poverty; that these causes must necessarily have produced great disturbance of the nutritive process; and that the circumstance of cod-liver oil having been taken by the women, in whom the deformity of the pelvis was observed, was probably a mere coincidence, and by no means the cause of the osteo-malacia.* This view is the more probable, seeing that this peculiar affection has not been observed in other districts, in which fish oil is largely used as a medicine.

Dr. *Delcour* has collected together a number of cases of genuine rachitis and of scrofulous disease of the bones of a very unfavourable character, in which the most marked benefit was unquestionably derived from the use of this remedy. The high authority of many of the reporters is a sufficient guarantee for the full accuracy of the statements, and we only regret that want of space prevents us from giving them here at length. When we mention that such a man as M.

---

* By referring to the number of the Medico-Chirurgical Review for October 1839, p. 549, in which will be found an account of M. *Hoebeke's* extraordinary success in Cæsarian operations, it will be perceived that we took a similar view of the subject.—*Rev.*

*Bretonneau* has been convinced from the results of his own experience of the efficacy of the remedy, we need say nothing more.

As to its effects in inveterate rheumatism, especially when this occurs in a lymphatic and weak habit of body, our author remarks: "There is not a practitioner at Verviers, where chronic rheumatism complicated with scrofula is exceedingly common, who has not had occasion to observe the admirable effects of cod-oil in such cases. By its use, a number of persons, who for a length of time had been almost paralytic and martyrs to protracted suffering, have been not only relieved, but perfectly restored to health. Like every other remedy, it does not succeed in all cases; but it has the advantage of being never hurtful. We again repeat that it is much more efficacious in weak than in robust plethoric constitutions."

M. *Tauflieb* has recorded in the Gazette Medicale for Nov. 1839, two most interesting cases, in which patients, who had been quite helpless and bedridden for several years in consequence of the swelling and stiffness of their joints, regained, after five or six months treatment, the complete use of their limbs.

Professors *Graefe* and *Ammon* have used the oil in several cases of obstinate rheumatic ophthalmia with decided advantage. Their report will be found in the Ophthalmological Journal of the latter for 1832. M. *Carron de Villards* speaks favourably of it as an application to specks and ulcers of the cornea, unaccompanied with inflammation; and the editor of the Archives de la Med. Belge has given a most favourable report of it in such cases, in the Annales d'Oculistique.

The dose of the cod or ray liver oil is from two to four table-spoonfuls for an adult, and as many tea-spoonfuls for children, in the course of 24 hours: the patient should close his nostrils, when he swallows it. To counteract the unpleasant eructations, a small portion of anisette, or of rum or brandy, may be taken immediately afterwards. If the mouth be previously gargled with any of these liqueurs, the taste of the oil will be much disguised. Dr. *Fehr* recommends the following formula for children: of cod-oil an ounce, of subcarbonate of potash one or two drachms, of oil of the calamus aromaticus three drops, and of syrup of orange-peel one ounce—one or two spoonfuls for a dose night and morning. Or an emulsion may be made by triturating the oil well with gum, sugar and water. When the stomach will not retain it, it may be administered in the form of enemata; and this mode of exhibition has succeeded in a good many cases.—*Archives de la Med. Belge, Juin* 1841.

*Remark.*—The use of cod and other fish oil is scarcely known as a remedy in this country, at least by medical men. We have indeed heard of cures being effected with it by extra-professional *doctors* in cases of obstinate rheumatism, and affections of the joints, &c., which had baffled the regular practitioner; and we see no good reason why the latter should not avail himself of a new remedy, when those in ordinary use fail, merely because it is a favourite with some empirics. There can be no doubt that fish oil must exert no inconsiderable effects on the system, independently of the iodine which it contains; its penetrating balsamic properties seem to be somewhat analogous to the class of the terebinthinate medicines, most of which are recognised to be most rapidly absorbed, and pervade every organ and part of the body. Those, who may wish to have more details on the subject, will do well to consult the memoir from which the preceding observations have been extracted.—*Rev.*

---

### Phthisis Pulmonalis, with a Fistulous Opening in the Parietes of the Chest.

The following case, exhibiting a very rare complication of phthisis, deserves the notice of the pathologist.

A man, 38 years of age and who for a length of time had been extremely subtect to attacks of catarrh, was seized in May, 1839, with pneumonia; this yielded to active treatment, but there remained behind a dry cough, and a feverish state, accompanied with frequent chills. In the beginning of July, a phlegmonous swelling made its appearance immediately below the right mamma; it gradually increased in size, and, as a fluctuation became distinctly perceptible, an opening was made into it and gave issue to an enormous quantity of a purulo-sanguineous fluid of a suffocating odour. This discharge continued night and morning usually to the amount of three of four ounces; the opening was situated between the fourth and fifth ribs.

For three weeks there was no reason to believe that a communication existed between the outward orifice and any of the bronchial tubes; but, on the 20th of August, it was observed for the first time that air escaped with a bubbling noise during the act of expiration, and when the patient coughed or spoke. It was easy to trace, by listening to the direction of the cavernous *souffle* which was very distinct, the course of the fistula inwardly. The pectoriloquy also was so loud that it seemed as if the patient spoke directly into the ear of the auscultator. Two months subsequently, a second opening between the fifth and sixth ribs was formed, and gave issue to a purulent discharge mixed with air. In the first week of December, the patient expectorated for the first time a small quantity of purulent sputa. There commenced also at this time occasional attacks of orthopnœa; but the breathing during the intervals was not much distressed: the emaciation was extreme. A remarkable feature of the case was an exceedingly constipated state of the bowels, with an almost voracious appetite. On the 9th of January, the fifth rib was nearly exposed for about two inches in extent, being covered only by a few pale granulations; the quantity of the discharge by the wound continued as before; but the expectoration had quite ceased. The denuded portion of the rib became necrosed, and was gradually detached in small pieces. The patient died exhausted on the first of April.

(We suppose that a dissection was not permitted, as there is no mention in the report of the appearances after death.)

*Remarks.*—It is doubtful whether the formation of the abscess in the parietes of the chest was the effect of the pulmonic lesion extending itself towards the surface, or whether it was not rather a simultaneous disease developed accidentally over the situation of the cavern in the lungs. The circumstance of no air being observed to escape for three weeks after the opening of the abscess may lead us to adopt the latter opinion.—*Archives de Medecine Belge.*

We observe that M. *Raciborski* recently exhibited to the Royal Academy a case in which a tuberculous excavation of the lungs communicated with a subcutaneous *foyer.* The disease, says that gentleman, seems to be confined to a point of the left lung over the fourth or fifth rib; the respiratory sound being normal over the whole of the front of the chest. Over the spine of the clavicle and near the root of the bronchi, a loud gurgling noise is heard, especially during the fits of coughing. The pulmonic abscess communicates with the subcutaneous cellular tissue at this part, and the skin there is observed to be distinctly lifted up during each fit of coughing. By applying the hand over the part not only may this rising of the skin be perceived, but a sensation of the displacement of a fluid may also be felt. Compression causes the swelling to disappear, and occasions a peculiar sound arising from the retrocession of the air and fluid. —*Rev.*

### M. Rayer on certain Lesions and Morbid Formations in the Kidneys.

In the last and present numbers of this Journal we have reviewed at considerable length the practical parts of M. *Rayer*'s important work on the diseases of the kidneys.

To complete the analysis, we have selected for insertion here some of the more rare lesions to which those organs are subject, in order that our readers may have a full summary of the three volumes.

We begin with—

*Renal Fistulæ* are the consequence of suppuration of the pelvis and calices of the kidney—induced, in the majority of cases, by the presence of impacted calculi,—when there is some obstacle to the passage of the pus along the ureters. The ulcer may open either into the extra-peritoneal cellular substance, or into the colon or duodenum, or into the cavity of the peritoneum, or even of the pleura, or it may make its way to the surface through the lumbar muscles, or in the groin.

The fistulæ are seldom numerous; in a few instances, however, M. *Rayer* has seen the posterior surface of the kidney, lodged in a vast purulent hollow, quite like a sieve, from the numbeı of perforations which it exhibited.

When the fistula opens outwardly, the purulent discharge is sometimes found to be mixed with urine. A similar occurrence has occasionally been witnessed, when the abscess has established a communication with any portion of the bowels.

In some cases, a renal fistula has been known to remain open for months or even years; and in others it has healed up very quickly, after the escape, spontaneous or by operation, of one or more calculi.

Besides the presence of calculi, wounds and contusions of the loins have been known to give rise to the formation of renal fistulæ. *Dessault* has recorded a case in which the disease seemed to have been induced by an impediment to the free discharge of urine from the urethra: the patient did well after the urethral obstruction was entirely removed. *Chopart* relates a case, in which a renal fistula, from which a calculus was extracted, communicated with an abscess in the corresponding groin. M. *Cruveilhier* met with a curious case of a woman who died from hectic fever, the cause of which could not be ascertained during life: on dissection the two kidneys, joined together, were found lodged in the pelvis, and contained a quantity of pus, part of which had made its way into the rectum. In a very few instances, renal fistulæ have been found to have communicated with the lungs; four such cases are related by M. *Rayer*. One of them occurred in a man, who, many years before, had been cut for the stone. He had frequent attacks of severe pain in the left loin, and the urine was sometimes strongly purulent. An attack of purulent expectoration came on very suddenly, with marked relief to the renal symptoms; but, as may be anticipated, he died soon afterwards. On dissection, a communication was traced between the pelvis of the kidney, which contained a quantity of pus and also a calculus, and the bronchi of the left lung.

### *Hydronephrosis, or Dropsy of the Kidneys.*

When the urine slowly accumulates in the kidneys, in consequence of some obstruction to the free escape of the urine into the bladder, or to its discharge by the urethra, it sometimes happens that the pelvis and calices gradually become more and more dilated, without any sensible inflammation of their parietes. Such a collection of a watery fluid, at first urinous and subsequently assuming a serous character, has been denominated hydro-nephrosis. The distention is at first limited to the pelvis and calices of the affected kidney; but gradually its parenchymatous substance becomes as soft and yielding as the coverings of a cyst. In some cases, one or both kidneys have been found transformed into enormous pouches, on the surface of which were observed little islands, so to speak, of the renal substance.

The openings of the calices into the pelvis of the kidney may become obstructed or obliterated, and give rise to dilatations, partial *hydronephroses*, or sorts of urinary cysts. This alteration is very rare in men; but common in cattle.

In the majority of cases, the lobular cysts in the kidneys of the ox have this origin. Occasionally they become exceedingly large, so as to even exceed the size of a child's head.

When the accumulation of fluid in the kidney becomes considerable, there is usually a more or less distinct and prominent swelling in the corresponding lumbar region. This sometimes gives the sensation to the finger of a distended intestine.

However strange it may appear, it is quite true that the abdomen has been known to become so immensely distended by watery distention of the kidneys, that the patient has actually been supposed to have been pregnant. In one case, the swelling formed by a distended kidney was mistaken for a dropsy of the ovarium.* From a comparison of the cases of dropsy of the kidney on record, it appears that both organs are more frequently affected at the same time with the disease than only one. M. *Piorry* shewed his pupils, in 1832, a kidney that had become so enlarged in dimensions that it measured a foot in length, and five or six inches across; the disease was the result of the ureters being obstructed by a calculus.

Cancer of the uterus seems to be a not unfrequent cause of obstruction of the ureters, and consequent hydro-nephrosis of the corresponding kidney.

### *Cysts of the Kidneys.*

These are small accidental vesicles or sacs which may contain not only a fluid, which is sometimes partially urinous, but also hydatids, &c. They are of not unfrequent occurrence, and may be classified in three groupes—simple cysts, acephalocystic, or hydatidic cysts, and urinary cysts. The first sort is much more frequently met with than the other two. They usually contain a slightly yellowish fluid, which is generally albuminous and somewhat saline. Occasionally the contents are more or less decidedly gelatinous or even still more consistent.

These cysts may be found in the cortical substance of the kidney, in the cellular tissue enveloping the renal vessels, or in the tubular substance of the organ: the former situation is the most common. These formations seem to be often induced by inflammation of the kidney. But in some cases vast numbers of them are developed without any antecedent disease. M. *Rayer* has represented in his Atlas one case in which the cysts were so numerous that the renal substance was completely disorganised. The same result, atrophy of the kidneys, has been induced by the formation of large cysts in the cellular tissue which invests the renal blood-vessels.

Cases of remarkable encysted degeneration of the kidneys have been recorded by *Hufeland, Howship, Bouillaud,* &c. In one instance, which occurred in a

---

* *Frank* alludes to a case in which the left kidney had become so enormously dilated, that upwards of sixty pounds of fluid was contained in the sac; the parenchymatous substance had entirely disappeared, and nothing but the enveloping membranes remained. The specimen is preserved in the Anatomical Museum at Vienna.

man who had suffered several times from nephritis, both kidneys were found on dissection to be as large as the head of a new-born child. The right one extended into the epigastrium behind the stomach, and descended below the cæcum; and the left one extended upwards to the diaphragm, and down as far as the iliac region. The surface of both kidneys was covered with numerous vesicles of various shapes and sizes; their contents being in some limpid, in others yellowish, and in a third set dark and semi-purulent. No trace remained of the cortical substance, and all that could be detected was a portion of the calices, which by their union formed the pelvis: these parts, as well as the ureters and the bladder, were perfectly sound, and contained no calculus.

The second kind of renal cysts, the hydatidic, are much more common in some of the lower animals, especially the sheep, than in man: in the former they are usually single, in the latter multiple. When these cysts have attained a large size, they have been known to occasion so distinct a swelling in the lumbar region as to be discoverable by the hand. The cysts usually contain a number of minute ones, which are either floating about in the fluid, or adhering to their walls. In the sheep, many of the cysts become at first encrusted with, and subsequently converted into chalky formations, these may be sometimes enucleated entire from the surrounding parenchyma of the affected organ. Occasionally the transformation is rather of an osseous than of a cretaceous character. The hydatidic cysts sometimes burst and open into the calices or pelvis or the kidney; and then some of the hydatids may be discharged with the urine.

When one or more hydatids have been passed in this way, should fresh pains in the renal region, or along the course of the ureters, come on, we may presume that new acephalocysts will be voided either alone or along with coagula of blood or with calculi. In a few very rare cases, the cysts have become inflamed, and have at length formed a communication outwardly in the loins.

The internal use of turpentine has seemed in some cases to have been useful in promoting the expulsion of the hydatiferous cysts.

Dr. *Weitenkapf* has published an account of a case which occurred a few years ago in his practice, and in which the patient passed on some occasions as many as 50 or 60 hydatids with the urine at a time; the hydatids were of that kind called *cystocerci:* some of them were alive when discharged. In a similar case, which is recorded in *Chopart's* work, the left kidney was found on dissection converted into a large membranous sac, which was filled with a number of hydatids of different sizes and forms. In the Bibliotheque Medicale for 1814 we find the following curious details of a case, in which an abscess formed in the loins, and discharged a number of hydatidiform cysts. A man, 68 years of age, had been long subject to wandering rheumatic pains, which at length fixed in the left thigh and leg. A small swelling made its appearance in the left groin; but for some years it gave little uneasiness to the patient. When it acquired a considerable size, it was accompanied with deep-seated pains, which appeared to extend to the left loin. In the course of a few days it disappeared, and a large abscess was formed in the lumbar region. For about a month this gave rise to severe pain; but at length it burst and gave issue to an enormous quantity of matter, in which were observed several bladder-like bodies, some of which were very small, and one or two as large as a pigeon's egg. For several successive days, a number of these vesicles were discharged from the wound in the loins. At the expiry of six weeks the wound was cicatrised, and the patient had quite recovered.

### *Worms in the Kidneys.*

Although frequent mistakes have been made by describing, as worms voided with the urine, portions of albumen and of coagulated blood, or the larvæ of insects which had been accidentally in the urinal at the time of making water, there is now no doubt that, in a few instances, worms have really been discharged from the urethra. Three kinds have been observed—the *strongylus gigas,* the *spiroptera hominis,* and the *dactylius aculeatus.* Besides these, intestinal worms have occasionally been known to make their way into the urinary passages, and to be voided with the urine.

The *strongylus* has been found not only in the human subject, but also in the kidneys of the dog, the wolf, the horse, and the seal. *Ruysch* says: "in renibus humanis semel eos me vidisse memini, quales in canum renibus longe frequentius occurrunt."

One of the most curious cases is related in the Journal of Medicine and Surgery for July 1758. A child, nine years of age, who had been cut for calculus four years before, was suddenly seized with retention of urine, accompanied with most severe pain in the right loin, numbness of the thigh on that side, hiccup, vomiting, &c. He was bled, put into a warm-bath, had an anodyne lavement, &c. Not more than a small cupful of thick turbid urine came away by the catheter. The active symptoms continuing, the bleeding, &c. required to be renewed; the urine was still very scanty and deep-coloured. The right loin now became puffy and swollen, and at length a feeling of fluctuation was indistinctly perceptible in the part; the patient having previously had repeated attacks of shivering. After the use of poultices had been continued for some time, a deep incision was made upon the most prominent part of the swelling: a large quantity of pus, mixed with sanguineous shreds, flowed from the opening. The discharge continued for some months; during several weeks it was tolerably healthy, but afterwards it became thin and ichorous. A variety of means were tried to improve its character, and to induce the healing of the wound; but every thing seemed to fail; and the surgeon recommended that nothing else should be done, except to keep it clean, and apply any mild application to it. Some months afterwards he was surprised to find that the wound had entirely healed, although the patient's general health was by no means re-established. But this state of things did not continue long; for again he was seized with retention of the urine, accompanied with excessive pains, and an insupportable sense of weight in the loins. On examining the wound, the surgeon felt a fluctuation upon pressure; an incision was made, and a quantity of purulent matter discharged, with immediate relief to all the distressing symptoms. Once more the wound healed; and some time afterwards a similar train of suffering supervened, and was relieved in a similar way. The urine was sometimes purulent, and almost always more or less filamentous. The fistulous ulcer had now continued, with occasional closures, for nearly three years, when the surgeon, suspecting that the excruciating pain which his patient suffered, and the frequently recurrent attacks of ischuria proceeded from the lodgment of a small calculus at the bottom of the wound, proposed that a deep incision should be made in hopes of extracting it. Before this was assented to, the mother of the boy was surprised to find one evening a living worm in the wound; she drew it out with her fingers, and fortunately preserved it. It was about five inches long, of a greyish colour, and as thick as a common quill. A second worm, rather shorter and smaller than the other, was extracted by the surgeon himself in the course of the following day. Two days later, the boy was suddenly seized with ischuria; and, as there was much distention and pain in the hypogastrium, it was suspected that the bladder must be full of urine. On attempting however to introduce a catheter, a complete obstruction was experienced at the cervix. After several ineffectual attempts to get it into the bladder, the boy was put into a warm bath; but, while in it, he was seized with such violent convulsions, that it was necessary to take him out. Judge of the surprise of the surgeon, who was about making another attempt to pass the instrument, when he observed at the opening of the urethra a foreign substance, which, upon being drawn out, proved to be a living worm, similar in size and appearance to the first one that had been taken from the wound. In the course of the following night, another worm was discharged from the urethra. From this time the ulcer in the loins began to discharge less and less, and gradually healed up. The urine, which now recovered a healthy appearance, occasionally contained filaments and membranous shreds, which were perhaps the debris of the cyst in which the worms had been contained. The boy was ultimately restored to perfect health.

Another remarkable case is related, in which an abscess made its appearance in the lumbar region and burst. It did not shew any tendency to heal, and the patient gradually became hectic and at length died. It is mentioned in one part of the report that, after taking some brisk purgatives, the woman had passed a dozen of worms—the kind is not stated. On examining the abdomen after death, it was found that the right kidney had become united to the liver; on cutting into it, there was discovered not only a large calculus imbedded in its substance, but also three living worms—each of them about three and a half inches long.

The number of worms, which were passed in the extraordinary case related by Mr. Lawrence, in the second volume of the Medico-Chirurgical Transactions, is estimated at between 800 and 1,000: they were of the sort which has been called *spiroptera* by *Bremser* and *Rudolphi.*

Mr. *Curling,* assistant-surgeon of the London Hospital, has recently published a case of a girl who voided from the urethra a number of entozootic worms, not hitherto described, with an account of the animals in the 22nd volume of the same work. Mr. C. proposes the name of *Dactylius aculeatus* for this species of worm.

### *Foreign Bodies and Irregularities in the Kidneys.*

Besides calculi, effused blood, and worms, foreign bodies admitted from without have occasionally been found in the substance of these viscera. A ball, or a piece of wadding or of cloth, has been extracted, either during the life or after the death of the patient; and, in a few rare instances, a small irregular mass, formed by one of the latter substances, has actually made its escape from the urethra, several weeks after the receipt of the musket-wound in the loins. On the other hand, an ear of corn, introduced by the urethra, has been known to find its way along the urinary passages and make its exit in the loins; and several cases have been recorded of needles, which had been swallowed, being voided with the urine.

There is sometimes a third or supernumerary kidney present; but a much more common anomaly is the existence of only one kidney. References to an immense number of such cases are given by M. *Rayer.* Both kidneys are sometimes altogether wanting in certain cases of monstrosity; and, if we could give credit to the accuracy of the examination that was made in the case related by M. *Moulon,* in the 17th volume of the Archives Generales de Medicine, we must believe that there was a complete absence of these organs in a girl who lived till she was fourteen years of age. There had been, during life, a constant oozing from the umbilicus of a fluid which had a very strong urinous smell. M. *Moulon* suggests that, in the absence of the urinary apparatus, the principles of this secretion were eliminated by the liver, and conveyed by the umbilical vein to the umbilicus, at which point they were discharged.

If this very extraordinary fact can be credited, we might find less difficulty in accounting for certain cases of *anuria* of several months' continuance, according to the testimony of several authors. Thus, in the Medical Journal that was edited by MM. *Corvisart, Leroux,* and *Boyer,* there is related an instance, in a young girl, of suppression of the urine which lasted for 17 months: the secretion returned after this period.

M. *Rayer* seems to think that the case narrated by M. *Moulon* was one of extrophia of the bladder, and that the kidneys, from being displaced, perhaps in the pelvis, were probably overlooked.

The two kidneys have often been found fused, so to speak, together, and forming a sort of horse-shoe viscus, situated right across the vertebræ.

We have already alluded to their occasional displacement within, or on the edge of, the cavity of the pelvis; where they might be mistaken for a tumor of the ovarium, uterus or rectum.

*Mobility of the Kidneys.*

There is a peculiar state of the kidneys in which these organs are capable of moving in different directions, as upwards towards the liver, or forwards and downwards. This state may become the cause of various distressing symptoms; more especially constant pains in the abdomen and the corresponding lower extremity, which are apt to be mistaken for colic or neuralgia. It is now a long time since I, says M. *Rayer*, first pointed out this peculiar affection to my colleagues at La Charité Hospital, MM. *Velpeau* and *Gerdy*, and shewed them several instances of it. Two cases occurred in the persons of medical men, who had long been suffering from pains in the right flank where they had felt a moveable tumor, which had greatly perplexed their friends as well as themselves.

M. *Rayer* candidly acknowledges, that references to this peculiar state of the kidneys are to be found in the writings of some of the older authors, and quotes a passage from the work of Riolan, published in 1682, in which there is a very accurate description of it,

He (M. *Rayer*) relates four cases.

In the first, the right kidney could be distinctly felt; it was so moveable that it could easily be pushed on near to the umbilicus, and then back under the edge of the liver. The woman complained of an uneasy dragging feeling in the loins and round the abdomen. She experienced considerable relief from a bandage applied firmly round the stomach.

In the second case, a tumor was felt under about the middle of the anterior edge of the liver. A variety of applications, including a moxa, had been tried, in hopes of dispersing it. M. *Rayer* attentively examined her, and, as the abdominal parietes were very lax, he could easily distinguish the size and shape of the tumor; it descended as low as nearly to the level of the umbilicus, but with gentle pressure it could be pushed upwards and backwards under the edge of the liver. He was convinced that it was in truth the right kidney.

An interesting case, which had occasioned great discrepancy of diagnosis among several medical men, is recorded by Professor Aberle of Saltzburg. A middle-aged man had long suffered much from dyspepsia and hypochondriasis. What, however, distressed him most, was a firm tumor, rather larger than a hen's egg, situated in the epigastric region; by firm pressure on the flanks, it became much more prominent and distinct.

He died from an attack of apoplexy; and, on dissection, it was found that the tumor was formed by the right kidney, which was so moveable that it could be readily pushed forwards almost as far as the umbilicus. There was no other abnormal character of the urinary organs.

M. *Rayer* assures us that mobility of the kidneys, usually the right one, is far from being uncommon, although hitherto the circumstance is not even known to almost any medical men.

It is much more frequently observed in women than in men; and is very often co-existent with an enlargement of the liver, or with some displacement either of some portion of intestine, or of the uterus. Repeated gestation, the lifting of heavy weights, &c. seem to have given rise to it in some instances.—*Traité des Maladies des Reins.*

